# Investigating the effects of cyclic topology on the performance of a plastic degrading enzyme for polyethylene terephthalate degradation

**DOI:** 10.1038/s41598-023-27780-4

**Published:** 2023-01-23

**Authors:** Heather C. Hayes, Louis Y. P. Luk

**Affiliations:** 1 School of Chemistry, Cardiff University Main Building, Park Place, Cardiff, CF10 3AT UK; 2 Cardiff Catalysis Institute, Cardiff University Main Building, Park Place, Cardiff, CF10 3AT UK

**Keywords:** Biotechnology, Chemical biology, Chemistry

## Abstract

Agitation is a commonly encountered stress for enzymes during all stages of production and application, but investigations that aim to improve their tolerance using topological engineering have yet to be reported. Here, the plastic-degrading enzyme *Is*PETase was cyclized in a range of topologies including a cyclic monomer, cyclic dimer and catenane using SpyTag/SpyCatcher technologies, and their tolerance towards different stresses including mechanical agitation was investigated. The cyclic dimer and catenane topologies were less susceptible to agitation-induced inactivation resulting in enhancement of polyethylene terephthalate (PET) degradation. While contrary to conventional belief, cyclic topologies did not improve tolerance of *Is*PETase towards heat or proteolytic treatment, the close proximity of active sites in the dimeric and catenane variants was found to enhance PET conversion into small soluble products. Together, these findings illustrate that it is worthwhile to explore the topology engineering of enzymes used in heterogeneous catalysis as it improves factors that are often overlooked in homogeneous catalysis studies.

## Introduction

Covalent cyclization modifies the topology of a protein leading to changes in biophysical properties that are of fundamental interest^1–3^. Existing techniques that are used to cyclize a polypeptide include head-to-tail cyclization, disulfide, or isopeptide bond formation between near terminal residues^4^. Alternative cyclic topologies include cyclic dimers and catenanes, formed by interlocking polypeptide chains^5–7^. Because cyclization restricts the number of conformational states accessible to the polypeptide backbone, population of the unfolded state is entropically disfavored and the tolerance of the cyclic protein towards elevated temperatures is expected to be enhanced, as illustrated in several homogeneous proteins such as β-lactamase, dihydrofolate reductase and GFP^8^. Rigidification of the polypeptide backbone may also improve the proteolytic resistance by restricting access of proteases^9,10^. On the other hand, the tolerance of cyclic proteins towards mechanical agitation (e.g. stirring, shaking, vortexing) upon cyclization is somewhat less explored, though a commonly encountered stress during all stages of protein production and application^11,12^.

**Figure 1:**
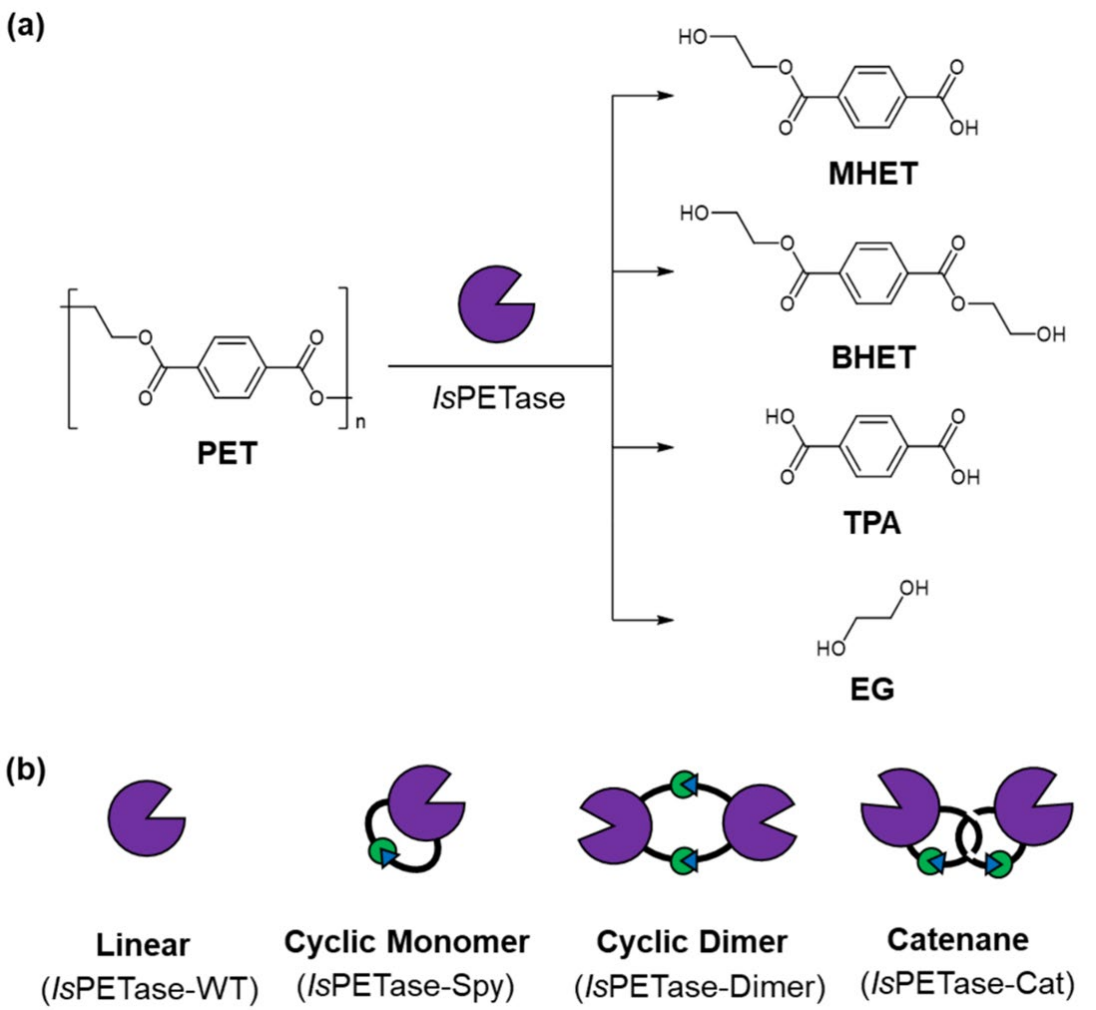
(**a**) The hydrolysis of PET catalyzed by *Is*PETase. (**b**) Cartoon representations of the linear and cyclic *Is*PETase variants.

In conventional heterogeneous systems, agitation is frequently used to increase mass transfer and hence the rate of reaction^13,14^, but it is a stress that induces enzyme inactivation through denaturation and aggregate formation^15–17^. Nevertheless, to the best of our knowledge, the use topological modification for improving the tolerance of an enzyme toward agitation has yet to be investigated^18^. Since protein stability was found to be both proteolytically and thermally stabilized when cyclized or catenated^19,20^, tolerance towards mechanical agitation may also be enhanced by a change in enzyme topology. An enzyme that could benefit from such stabilization is *Is*PETase which is viewed as a promising candidate for the recycling of polyesters^21–23^. First isolated from the polyethylene terephthalate (PET)-metabolizing bacterium *Ideonella sakaiensis*, *Is*PETase catalyzes the hydrolysis of PET, producing mono(2-hydroxyethyl) terephthalic acid (MHET) as the major product, as well as bis(2-hydroxyethyl) terephthalic acid (BHET), terephthalic acid (TPA) and ethylene glycol (EG) as minor products (Fig. 1a)^24–26^. Initial studies demonstrated that *Is*PETase is selective for PET substrates (compared to aliphatic esters) and kinetically outperforms other known PET-degrading enzymes under ambient conditions^24^. However, the utility of wild type *Is*PETase (*Is*PETase-WT) is limited by its low stability^27^, although a number of *Is*PETase variants with enhanced stabilities and activities have since been reported. The majority of these approaches relied upon rational design strategies^28–30^; yet, the most dramatic improvements to the stability of *Is*PETase to date have been provided by computation design and machine learning methods^31,32^, as well as a recent laboratory evolutionary approach^33^. Indeed, the thermally stable variant DuraPETase, created using a computational-based “greedy accumulated strategy for protein engineering (GRAPE)” strategy, exhibited a 300-fold enhancement in degradation activity towards a semicrystalline PET film compared to *Is*PETase-WT^31^, though its tolerance to agitation has not been examined. While this manuscript was in preparation, a patent for a cyclic variant of *Is*PETase was also filed^34^. Nevertheless, the tolerance of the cyclic *Is*PETase towards proteolysis and agitation as well as the actual activity towards PET degradation were not reported. Accordingly, we conducted these analyses on various topologically engineered *Is*PETases.

In the present study, three different cyclic topologies of *Is*PETase were prepared using the SpyTag/SpyCatcher isopeptide bond formation approach, including a cyclic monomer (*Is*PETase-Spy), a cyclic dimer (*Is*PETase-Dimer) and a catenane (*Is*PETase-Cat) (Fig. 1b). In contrast to much of the published literature and recent patent, cyclization of the *Is*PETase did not enhance the thermal or proteolytic stability of the enzyme. Instead, dimerization and catenation demonstrated moderate improvement to tolerance of mechanical agitation. These variants were also found to have enhanced PET degradation activity, likely because the active sites are placed in close proximity. To further examine the effects of topology on enzymatic PET degradation, cyclization of the thermally stable DuraPETase mutant was investigated. Interestingly, topological modification of this computationally redesigned enzyme did not aid its PET degradation performance or stability towards agitation, suggesting that cyclization affects enzyme performance in a case-dependent manner.

## Results and discussion

### Production of cyclic *Is*PETase

**Figure 2:**
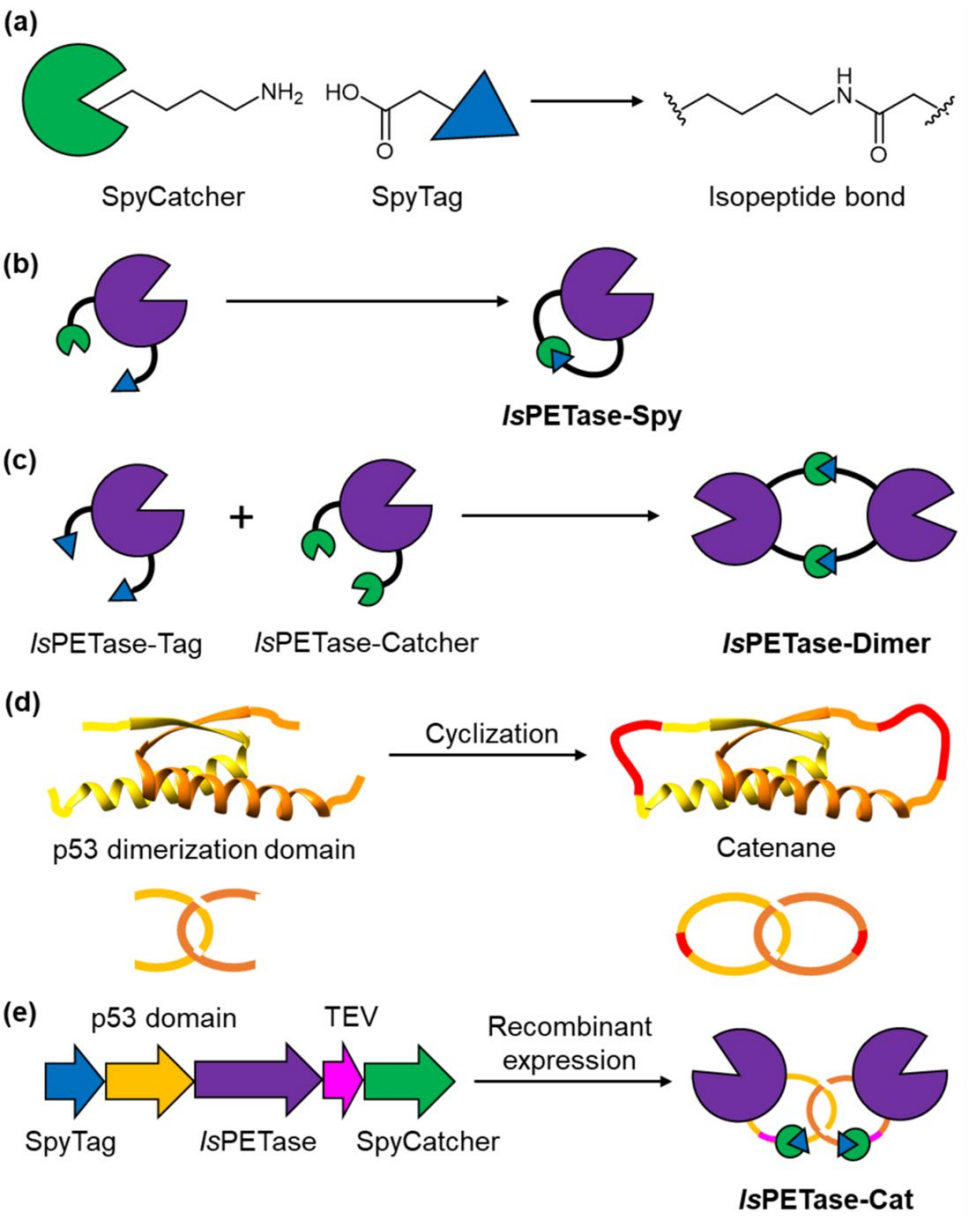
(**a**) Isopeptide bond formation between Asp and Lys on SpyTag and SpyCatcher, respectively. (**b**) Intramolecular isopeptide formation generating the *Is*PETase-Spy cyclic monomer. (**c**) Intermolecular isopeptide bond formation between *Is*PETase-Tag and *Is*PETase-Catcher producing the *Is*PETase-Dimer cyclic dimer. (**d**) Catenane formation by cyclization of the p53 dimerization domain (PDB 4D1L). (**e**) Diagrams of the *Is*PETase-Cat gene construct and protein catenane.

The different cyclic topologies of *Is*PETase were prepared using SpyTag/SpyCatcher, which yields an isopeptide bond between the reactive Asp and Lys residues (Fig. 2a)^35^. This peptide-protein reactive pair can be genetically incorporated at the termini of the protein of interest without the need for any post-translational modifications. In addition, isopeptide bond formation is spontaneous and largely independent of the protein used. Together, these features make the SpyTag/SpyCatcher technology a versatile and reliable cyclization approach^36^. It should be noted that the active site of *Is*PETase, containing the Ser-His-Asp catalytic triad, is located distally from the termini (Fig. S1)^25,26^. Consequently, the presence of the SpyTag/SpyCatcher cyclization machinery would not be expected to interfere with the access of PET to the substrate binding pocket. Moreover, the termini were extended using linker sequences to further limit disruption to the tertiary structure of the enzyme (see SI).

In the cyclic monomer enzyme, the SpyTag peptide was positioned at the C-terminus of the *Is*PETase sequence, while a circularly permutated variant of SpyCatcher (SpyCatcher-N^TEV^)^37^ was incorporated at the N-terminus. This version of SpyCatcher was used as it contains a cleavable TEV protease recognition site which allows for removal of a large portion of the SpyCatcher fragment after cyclisation has taken place, leading to a reduction in the size of the scar at the ligation site. Upon recombinant expression, the desired mono-cyclic enzyme was generated by intramolecular isopeptide bond formation (Fig. 2b). To produce the cyclic dimer, two different *Is*PETase monomers were generated. In one of these monomers SpyTag sequences were incorporated at both termini, whereas in the other monomer SpyCatcher fragments (without the TEV protease cleavage site) were positioned at the termini. Consequently, the monomers remained in their unreacted linear forms until mixed with their reactive partner, whereupon intermolecular isopeptide bond formation produced the desired cyclic dimer (Fig. 2c).

For the production of the *Is*PETase catenane variant, a dimer mutant of the tetramerization domain of the tumor suppressor protein p53 was employed^38^. It consists of two intertwined polypeptide chains (Fig. 2d). Upon intramolecular cyclization of the separate polypeptide chains, a catenane is produced^39^. In the *Is*PETase-Cat construct, the *Is*PETase sequence was positioned in between the p53 dimerization domain and SpyCatcher sequences at the N- and C-termini, respectively. The SpyTag sequence was incorporated at the N-terminal of the p53 dimerization domain (Fig. 2e)^19,20^. A TEV protease recognition sequence was also incorporated in between the *Is*PETase and SpyCatcher sequences to aid catenane characterization as described below. In addition, a linear control was generated by mutation of the reactive Asp in the SpyTag sequence (*Is*PETase-Cat^D7A^), and mono-cyclic control was prepared by modification of the p53 domain (*Is*PETase-Cat^K19P^).

**Figure 3:**
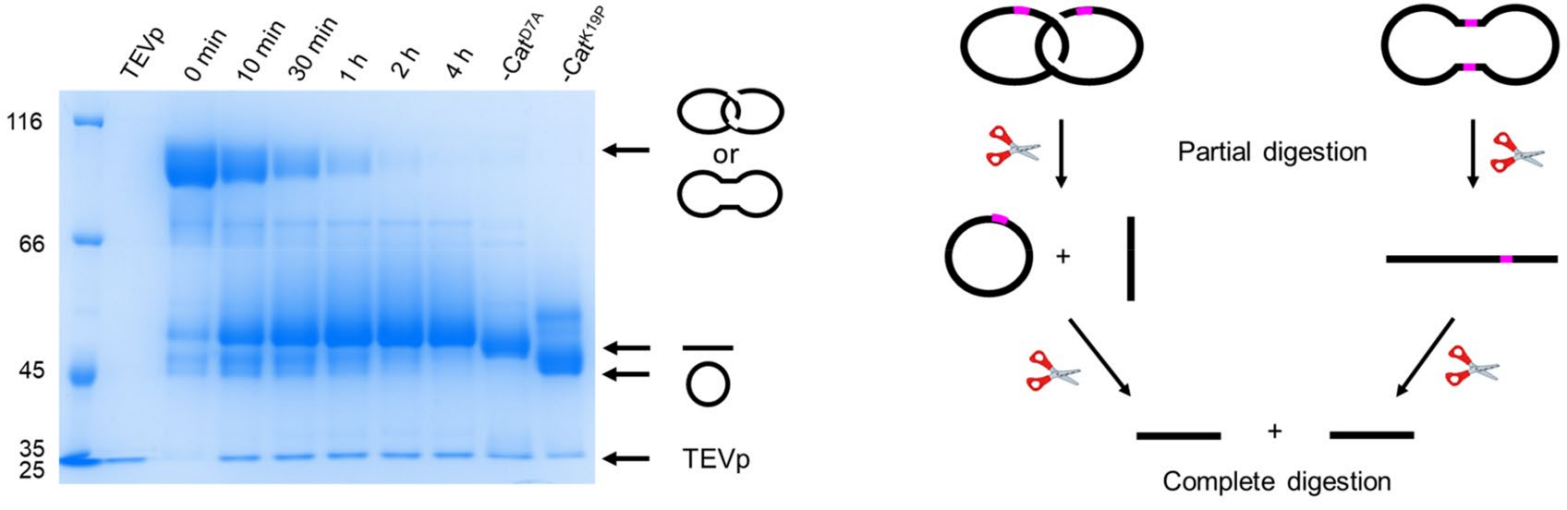
SDS-PAGE of the TEV protease (TEVp) digestion of *Is*PETase-Cat and a diagram of the expected partial and complete digestion products of a catenane and cyclic dimer (see Fig. S2 for the original gel).

To confirm that the *Is*PETase-Cat product was a catenane, rather than a cyclic dimer of the same molecular weight, analysis of the sample was carried out using TEV protease digestion. Partial digestion of the catenane would produce a mixture of linear and cyclic monomers, whereas the cyclic dimer would produce the linear dimer. SDS-PAGE analysis of the *Is*PETase-Cat partial digestion products revealed that the desired catenane was present in the sample (Fig. 3). In addition to the catenane, a significant quantity of cyclic monomer side product was also observed, suggesting premature cyclization during recombinant gene expression. This could not be completely removed by size exclusion chromatography (SEC), likely due to the formation of weakly associated dimers in solution^20^.

### Thermal stability

The secondary structures of the linear and cyclic *Is*PETase variants were analyzed using circular dichroism (CD) spectroscopy (Fig. S3). To determine the thermal stabilities of the linear and cyclized *Is*PETase variants, the unfolding of each protein between 10 and 90 °C was followed by using the CD signal at 222 nm. The estimated protein melting temperature (T_m_) for *Is*PETase-WT, -Spy, and -Dimer ranged between 42 and 45 °C, indicating that thermal stabilization of *Is*PETase was not achieved by cyclization (Fig. S4; Table S1). For *Is*PETase-Cat a T_m_ of 41.4 °C was measured, but this value should be interpreted with caution due to the presence of the cyclic monomer impurity. Nevertheless, the lack of change was somewhat unexpected, as the few protein catenanes that have previously reported exhibited improved stability compared to both their linear and cyclic counterparts^6,19,20^.

Cyclization can result in destabilization due to protein distortion^40^. As a result, extension of the connector would be expected to relieve the strain leading to an increase in thermal stability. However, increasing the number of amino acid residues in the linker between the *Is*PETase sequence and SpyTag/SpyCatcher-N^TEV^ cyclization machinery did not result in a significant improvement to the T_m_ of *Is*PETase-Spy (see *Is*PETase-Spy^+5^ and -Spy^+10^, Table S1). In addition, the linear control of *Is*PETase-Spy, in which the Asp residue responsible for isopeptide bond formation the SpyTag is mutated to Ala (*Is*PETase-Spy^D7A^), was found to be mildly destabilized compared to both the linear WT and cyclized enzymes with a T_m_ of 40.4 °C.

### Proteolytic stability

**Figure 4:**
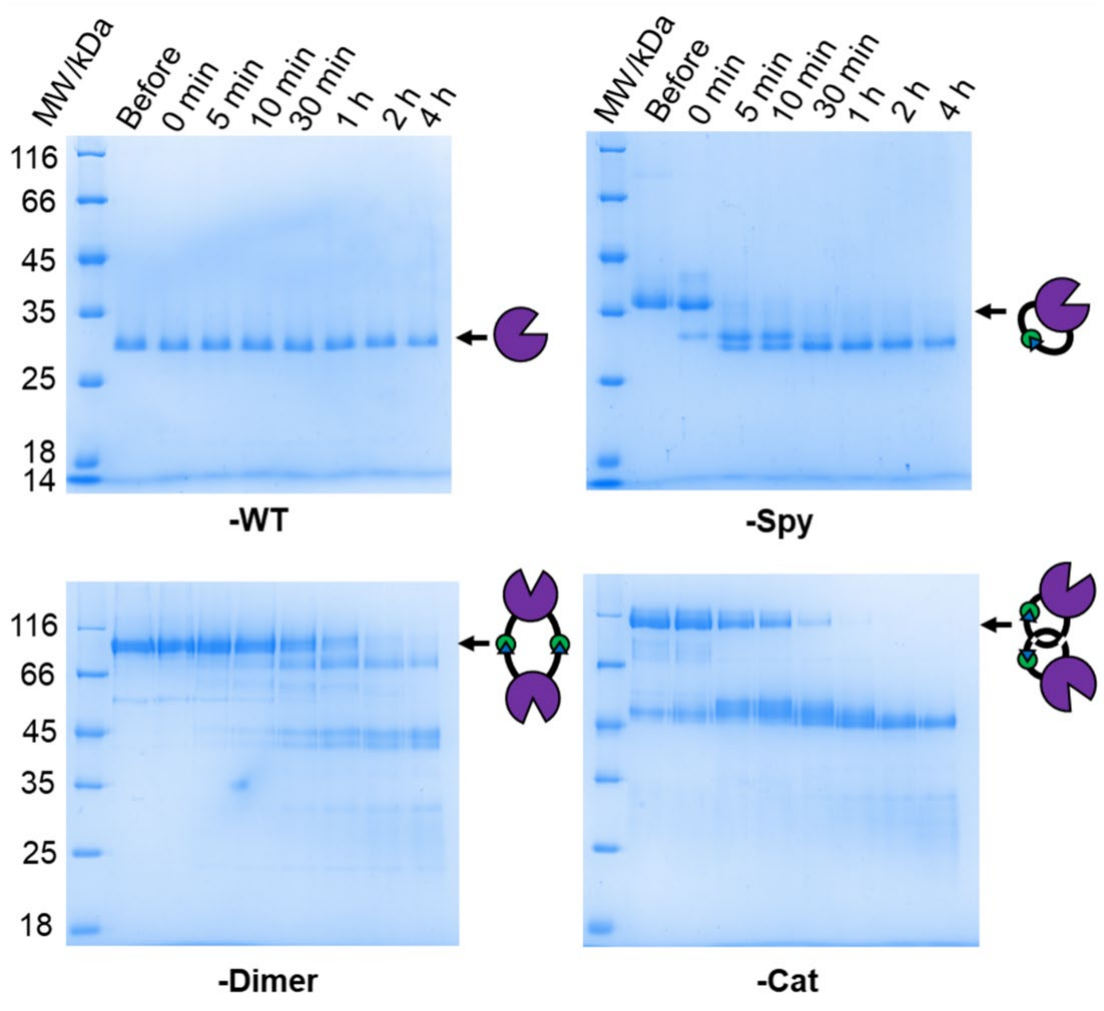
SDS-PAGE analysis of trypsin digest of the linear and cyclic *Is*PETase variants at 30 °C. 50 mM Na_2_HPO_4_ (pH 8.0) and 100 mM NaCl buffer was used with a 1:100 molar ratio of trypsin-to-*Is*PETase (see Figure S7 for the original gels).

To further compare the effect of topology on the *Is*PETase variants, trypsin digest was employed. Figure 4 shows the SDS-PAGE analyses of the trypsin digest of the linear and cyclic variants performed at 30 °C. The progress of the reaction was monitored by taking samples at timed intervals for up to 4 h. At 30 °C, *Is*PETase-WT appeared to be resistant to trypsin digest, with no observable difference in the size or location of the main band corresponding to the enzyme after 4 h. To our surprise, all the cyclized variants were found to be more sensitive to proteolytic digestion. The most rapid digestion was observed for *Is*PETase-Spy, with the majority of the main protein band digested in less than 5 min at 30 °C. *Is*PETase-Dimer was the cyclic variant most resistant to proteolytic digestion, though it was still almost completely digested after about 1 h. *Is*PETase-Cat was mostly digested after 30 min. As temperature was increased (40 and 50 °C, Fig. S5), shorter amounts of time were needed to digest the variants likely due to (i) the increased activity of trypsin; and/or (ii) the increased unfolding of *Is*PETase whereby protease cleavage sequences are exposed.

On closer inspection, the primary partial degradation product of *Is*PETase-Spy has a molecular weight similar to that of the linear WT enzyme, which demonstrated resistance to digestion (at 30 °C for 4 h). This suggested that the *Is*PETase sequence has high proteolytic stability towards trypsin, whereas the SpyTag/SpyCatcher(-N^TEV^) complex is prone to digestion. Moreover, the *Is*PETase-Spy^D7A^ linear control was rapidly digested (< 5 min at 30 °C, Fig. S6). This contrasts with previous literature reports in which intramolecular isopeptide bonds have been demonstrated to confer increased resistance to proteolytic digestion^41,42^.

### Residual activity

**Figure 5:**
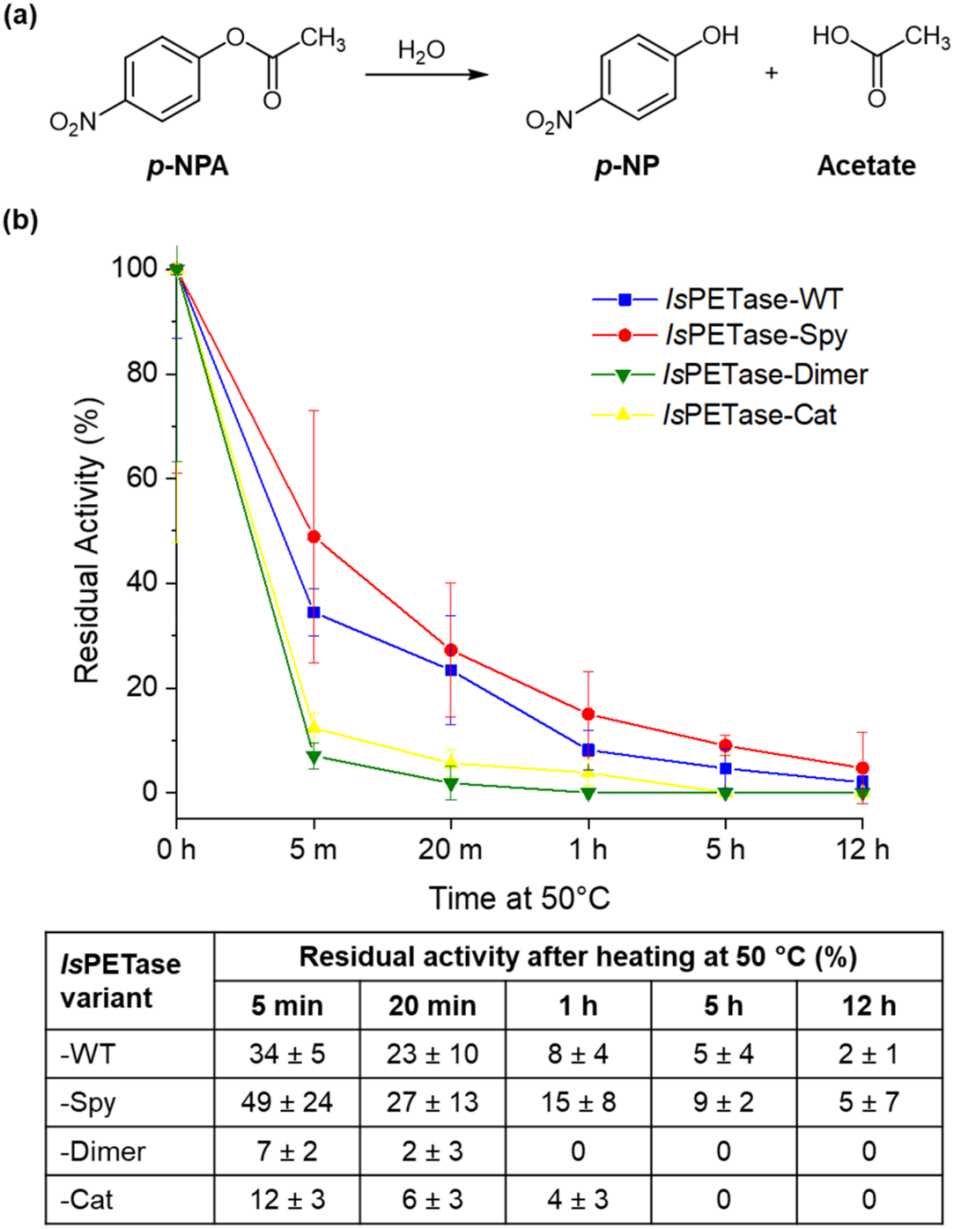
(**a**) *p*-NPA hydrolysis producing *p*-NP. (**b**) Percentage residual activities of *Is*PETse-WT, -Spy, -Dimer, and -Cat after heating for increasing lengths of time at 50 °C. Error bars represent the 95% confidence interval.

The esterase activities of the *Is*PETase variants were compared spectrophotometrically by assessing the rate of hydrolysis of *p*-nitrophenol acetate (*p*-NPA) (Fig. 5a)^43^. The increase in absorbance at 405 nm correlates with the release of the deprotonated *p*-nitrophenyl (*p*-NP) product (ε_405_ = 18,400 M^−1^ cm^−1^)^44^. The reactions were performed at 30 °C in a phosphate buffer at pH 7.5. The concentration of the monomeric enzymes (*Is*PETase-WT and -Spy) was 10 nM. Meanwhile, 5 nM of the dimeric enzymes (*Is*PETase-Dimer and -Cat) were used, taking into consideration that each molecule contained two active sites. During the calculation of kinetic parameters, the enzyme concentrations of the dimeric species were adjusted to reflect the active site concentration.

The estimated *K*_m_ value (2.7 ± 0.3 mM) determined for *Is*PETase-WT was similar to that previously reported in the literature for *p*-NPA hydrolysis (2.6 ± 0.3 mM)^45^. Therefore, the V_max_value extrapolated was assumed to be of a reasonable estimate. After fitting of the Michaelis–Menten model (Fig. S8), the estimated catalytic efficiencies (*k*_cat_/*K*_m_) calculated for all *Is*PETase variants was around 2 × 10^4^ M^−1^ s^−1^, suggesting that there was no significant catalytic difference among the four enzymes at 30 °C (Table S2).

Residual activity refers to esterase activity remaining after treatment that causes inhibition. Accordingly, the more tolerant the enzyme is towards the inhibiting conditions, the more residual activity it would exhibit. Here, the *Is*PETase variants were heated for increasing amounts of time at 50 °C followed by centrifugation. Subsequently, the activity of the enzyme remaining in solution was determined by assessing the extent of *p*-NPA hydrolysis at 30 °C (Fig. 5b; Fig. S9). *Is*PETase-WT and -Spy displayed the highest residual activities throughout, with both enzymes retaining partial activity even after heating for 12 h. Of the two enzymes, the cyclic monomer had the largest mean residual activity at each time point tested, though they were not found to be significantly different at the 95% confidence interval. In contrast, the activities of both *Is*PETase-Cat and -Dimer dropped by around 90% after only 5 min of heating, and were completely deactivated within 1 h of heat treatment.

During the preparation of this manuscript, a patent for an *Is*PETase variant cyclized by SpyTag and a non-circularly permutated SpyCatcher that lacks a TEV recognition site was reported^34^. Interestingly, this version of the cyclized enzyme was reported to retain ~ 70% of its *p*-NPA hydrolysis activity after heating for 10 min at 50 °C and up to 35% activity after heating at 90 °C. The linear WT enzyme employed in the patent retained only 10% activity after 10 min at 50 °C, whereas 23% conserved activity was conserved by *Is*PETase-WT after 20 min of incubation at the same temperature in this work. These discrepancies in residual activity could result from differences in (i) the cyclic enzyme constructs used (Table S3), and/or (ii) the experimental procedure used to measure the residual activity. Furthermore, the patent did not evaluate the performance of the cyclic enzyme for PET degradation under mechanical agitation (see below). As such, a direct comparison between the results of the patent and those reported here are not possible and will be the subject of a future study.

### Heat-induced aggregation

**Figure 6:**
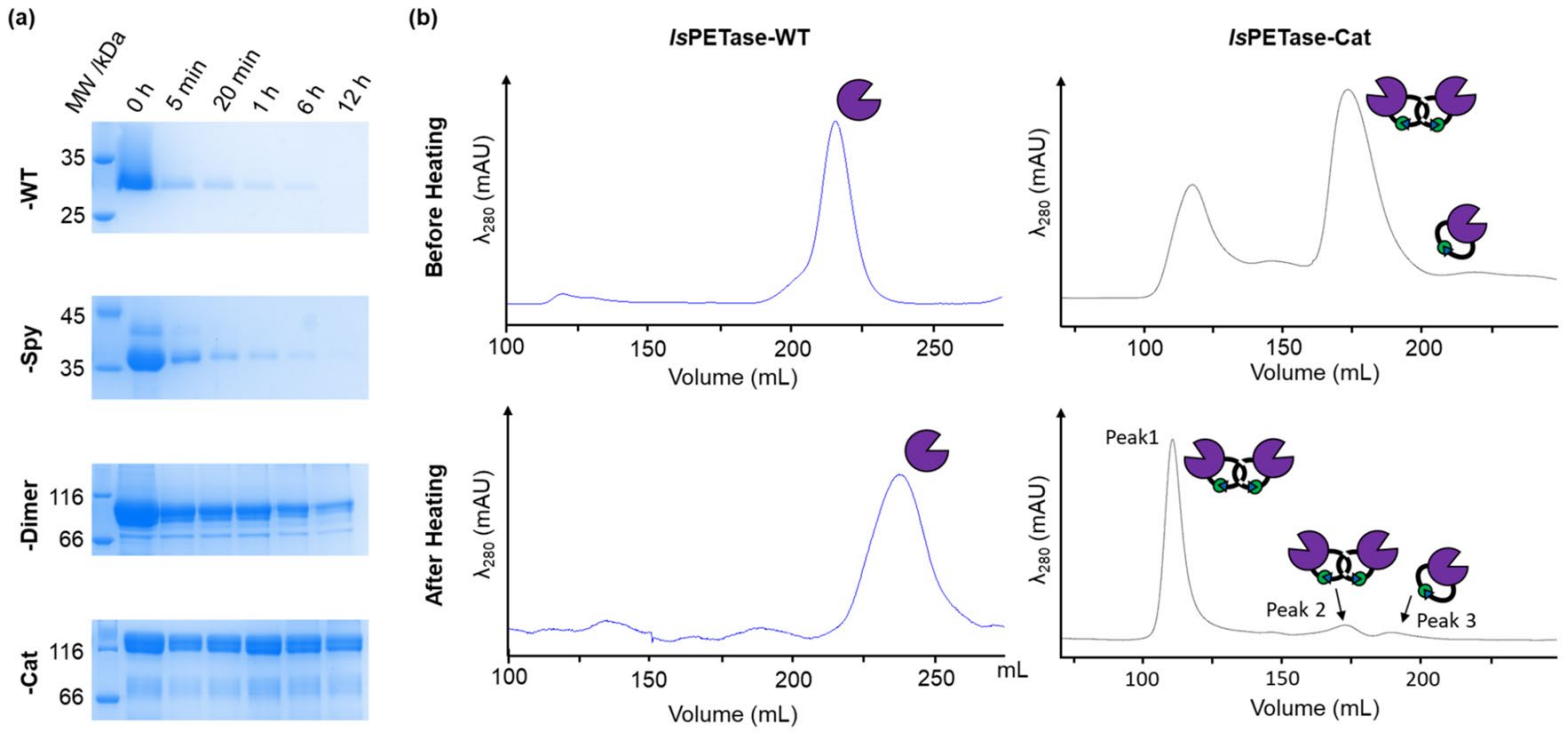
Analyses of *Is*PETase sample supernatant after heat treatment and centrifugation. (**a**) SDS-PAGE analysis of the linear and cyclic *Is*PETase variants after heating at 50 °C for increasing lengths of time (see Figure S10b for the original gels). (**b**) SEC chromatograms of *Is*PETase-WT and -Cat before and after heating at 50 °C for 5 min.

After heat treatment and centrifugation of the *Is*PETase samples, the supernatant was also analyzed by SDS-PAGE (Fig. 6a). For the monomeric samples, the concentration of enzyme remaining in solution clearly decreased with time, likely due to precipitation. This indicated that enzyme not lost from solution was partially inactivated leading to a drop in residual activity. On the other hand, the dimeric enzymes were retained in solution for longer than the monomeric samples, but there was a significant drop in residual activity. This suggested that the dimeric enzymes were less able to recover an active conformation after heat treatment despite not being precipitated.

Size exclusion chromatography (SEC) was employed to investigate how heating affected the structures of the monomeric and dimeric enzymes remaining in solution (Fig. 6b; Fig. S10a). For the monomeric enzymes, elution from the column occurred slightly later than for the fully folded protein (i.e. before heating). Heating would be expected to result in protein unfolding, leading to a less compact structure and a reduced retention volume^46^. However, hydrophobic residues exposed in the unfolded protein species could be interacting with the column resin leading to the increased retention observed^47,48^. Meanwhile, the chromatogram of the catenane after heating at 50 °C showed one major peak (Peak 1) and two smaller peaks (Peak 2 and 3). SDS-PAGE analysis found that the proteins eluted in Peaks 1 and 2 had molecular weights corresponding to that expected for *Is*PETase-Cat. Meanwhile, Peak 3 had a molecular weight corresponding to the cyclic monomer contaminant. Before heating, *Is*PETase-Cat was eluted at the same volume as Peak 2. Therefore, the large decrease in the elution volume of *Is*PETase-Cat is suggestive of the formation of aggregates, which are eluted in the void volume due to their large size. It is likely that this rapid aggregate formation correlates with the large decrease in residual activity observed for the dimeric enzymes.

### PET degradation

The PET degrading activities of the linear and cyclized *Is*PETase variants were directly assessed by using commercially available PET powder as the substrate. A bulk absorbance-based assay was employed as a means of measuring the release of soluble degradation products^49^. To evaluate the stability of the different *Is*PETase variants towards agitation, the assay was performed at increasing shaking speeds and increasing temperatures. The course of the degradation reaction was followed by taking samples at selected time points during the 24 h of incubation (Table S4–6).

**Figure 7:**
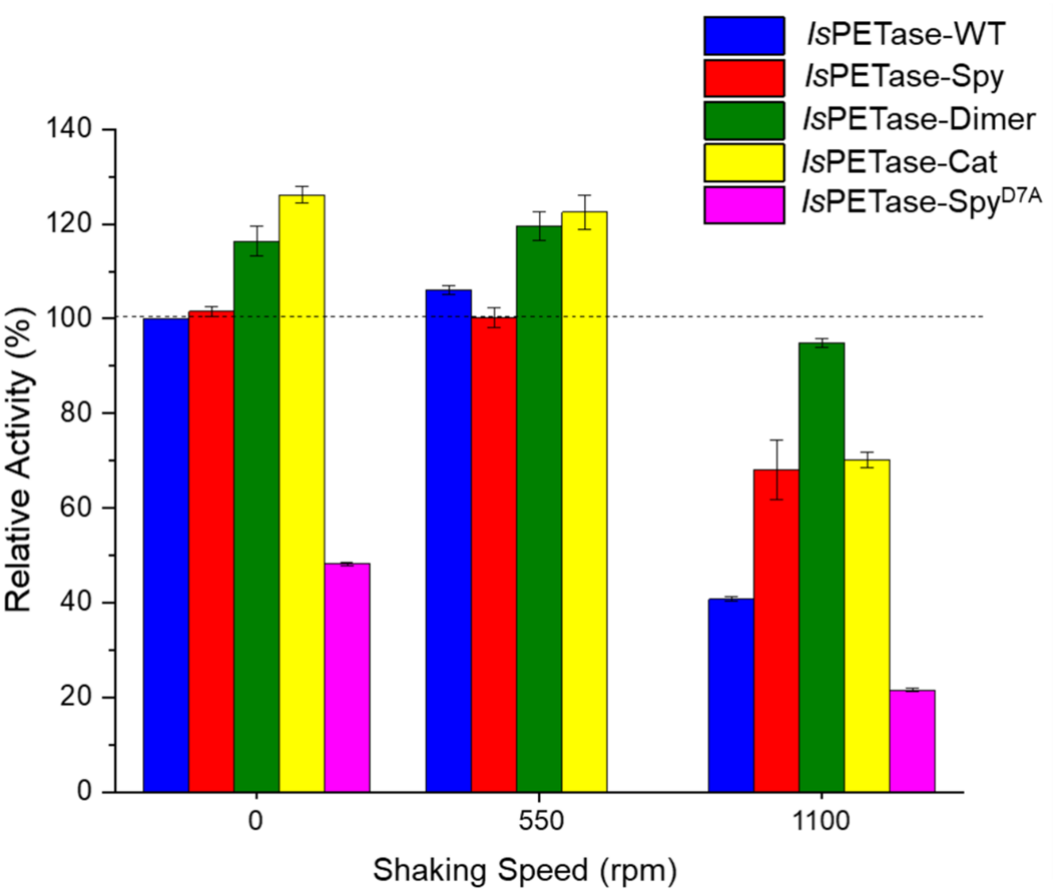
Relative PET degrading activity of the linear and cyclic *Is*PETase variants at 30 °C as a percentage of the concentration of soluble degradation products released by *Is*PETase-WT (i.e. 395 ± 16 µM) after incubation with 7.5 mg/mL PET for 24 h at 30 °C, 0 rpm. Error bars represent the standard deviation from the mean.

The effect of agitation on the activities of the linear and cyclic *Is*PETase variants were initially examined by assessing PET degradation activity at 30 °C for 24 h (Fig. 7). Notably, all variants exhibited the greatest activity when incubation was carried out at 0 or 550 rpm. Under these optimal conditions, the dimeric variants outperformed the monomeric enzymes producing 16–26% more soluble degradation products. A plausible explanation is that the close proximity of the dimeric enzymes’ active sites leads to an increased probability of substrate binding and product release into solution. The use of vigorous agitation (1100 rpm) resulted in a reduced yield of soluble degradation products released by all variants. Nevertheless, the cyclic variants still displayed improved activity compared to *Is*PETase-WT. In particular, *Is*PETase-Dimer maintained 80% of its activity compared to at 0 rpm followed by IsPETase-Cat and -Spy (~ 70%), whereas *Is*PETase-WT retained only 40%. This decrease in activity could indicate sensitivity of the enzymes towards agitation. Alternatively, the lower soluble degradation product yield observed for all variants could also be suggestive of ineffective mass transfer at the solid–liquid interface due to the increased force of agitation, resulting in reduced binding of the enzyme to the substrate^50,51^. However, the A_240_ reading suggested that the amount of soluble degradation products plateaued at different time points, which are inversely correlated to the extent of both agitation and temperature (Figs. S11–13). Since enzymes exhibiting prolonged activity produced a higher yield of degradation products, the results suggest that the cyclic variants are better at withstanding the stresses caused by increased agitation.

The activity of the *Is*PETase variants was also influenced by temperature, with fewer soluble degradation products released by all variants at 40 and 50 °C (Fig. S14). However, at these increased temperatures the extent of PET degradation displayed by the enzymes followed a slightly different trend regarding agitation speed. When vigorous shaking (1100 rpm) was employed at 40 °C, the monomeric variants exhibited reduced PET degradation. Most notably, a tenfold drop in PET degradation activity was observed for *Is*PETase-Spy compared to the reaction performed at 30 °C at 1100 rpm. In contrast, the extent of PET degradation achieved by the dimeric variants was less affected by the increased agitation speed. *Is*PETase-Dimer generated 10% more soluble degradation products compared to at 0 rpm at the same temperature. Furthermore, when temperature increased to 50 °C, the activity of both linear and cyclic variants was found to be improved upon agitation (1100 rpm). Indeed, all the enzymes generated roughly double the soluble degradation products compared to at 0 rpm. It should be noted that the linear *Is*PETase-Spy^D7A^ control generally showed reduced PET degrading activity compared to both the linear WT and cyclic enzymes. This suggests that the differences in the activity observed between the linear and cyclic variants resulted from topology and not just the presence of the SpyTag/SpyCatcher(-N^TEV^) sequences.

### DuraPETase

**Figure 8:**
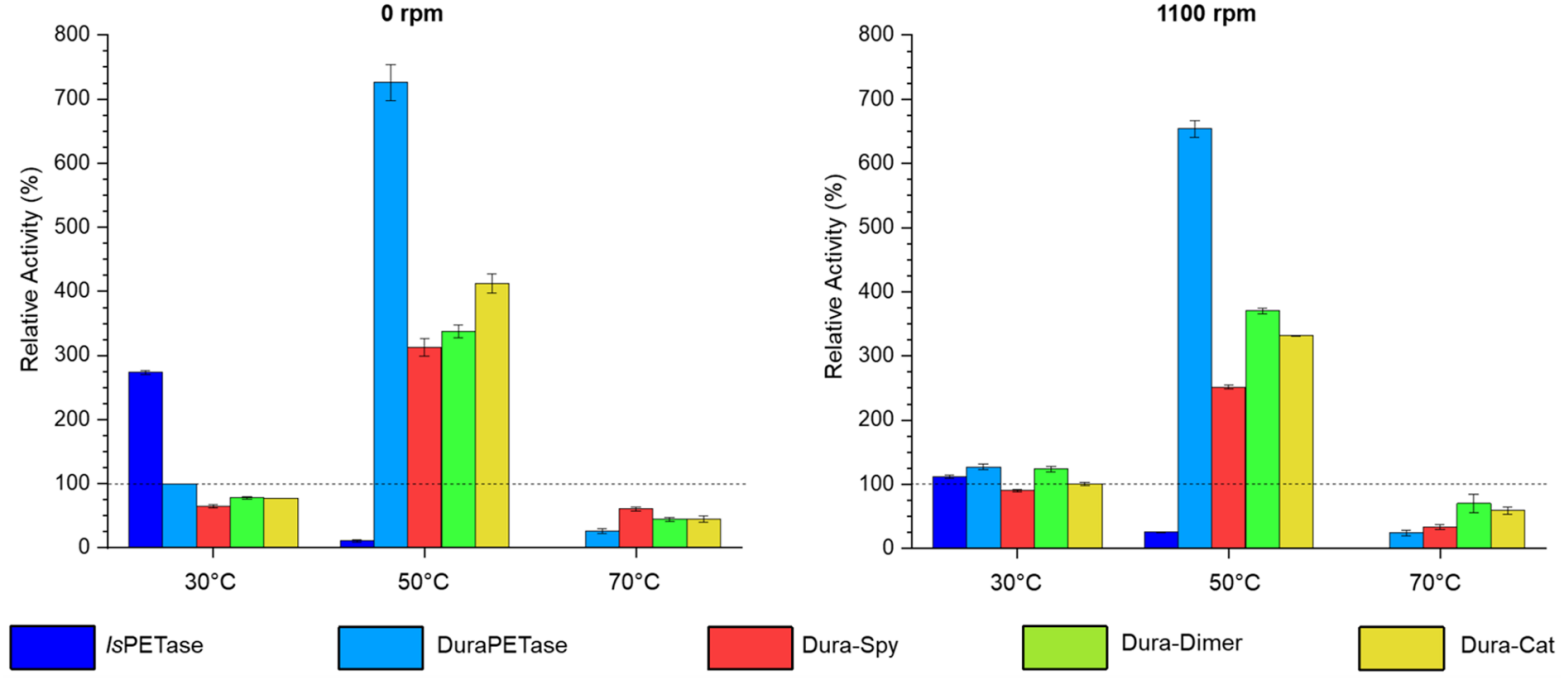
Relative PET degrading activity of the linear and cyclic DuraPETase variants as a percentage of the concentration of soluble degradation products released by DuraPETase after incubation with 7.5 mg/mL PET for 24 h at 30 °C, 0 rpm. Error bars represent the standard deviation from the mean.

The analysis above indicates that dimerization of *Is*PETase is beneficial for PET degradation most likely due to (i) the increased the probability of enzyme–substrate complex formation, and (ii) protection of the enzyme from agitation-induced inactivation. The general applicability of this concept was further investigated by modifying the topology of the thermally stable variant DuraPETase (T_m_ = 77 °C)^31^. The cyclic monomer (Dura-Spy), cyclic dimer (Dura-Dimer), and catenane (Dura-Cat) variants of DuraPETase were constructed using SpyTag/SpyCatcher(-N^TEV^) as previously described in this work. PET degrading activity was evaluated with and without agitation (1100 and 0 rpm) using the bulk absorbance assay at 30, 50 and 70 °C, which is roughly the glass transition temperature (T_g_) of PET (Fig. 8; Figs. S15–16; Tables S7–8). The yield of soluble degradation products generated by DuraPETase in the absence of agitation was roughly 700% more than was released by *Is*PETase after 24 h at 50 °C. Under these conditions, the linear DuraPETase outperformed all the cyclic variants, generating approximately double the soluble degradation products. However, at 70 °C, above the known optimal reaction temperature of DuraPETase (60 °C)^52^, all four DuraPETase variants exhibited minimal degradation activity. Furthermore, contrary to the observations made for *Is*PETase, dimerization of DuraPETase did not improve activity towards heat treatment or agitation. This could be because the properties of DuraPETase were already optimized for efficient PET degradation during the computational redesign process.

## Conclusions

To date, the majority of protein cyclization studies reported have concentrated predominantly on homogenous proteins and enzymes, with improvements in thermal and/or proteolytic stability often reported. In contrast to the literature, this work illustrates that the cyclization of *Is*PETase does not result in enhanced stability at elevated temperatures, but there is measurable improvement toward agitation upon cyclic dimerization and catenation. This demonstrates that changes to the topology of a heterogenous enzyme can influence its ability to withstand agitation-related stresses and could be beneficial for the enzyme catalysis of interfacial reactions. Furthermore, the close proximity of active sites in the dimeric variants may enhance the enzymatic conversion of PET into smaller soluble products. Hence, future work will aim to better understand the effects of topology engineering on the activity of heterogeneous enzymes in the presence of mechanical agitation, comparing the different variants of isopeptide bond forming complexes and other cyclisation approaches (e.g. intein-mediated ligation), with the aim improving the stability of *Is*PETase and other plastic-degrading biocatalysts.

## Materials and methods

### Materials

All reagents were purchased from Fisher Scientific®, unless stated otherwise. Custom oligonucleotides were purchased from Merck Sigma Aldrich. PrimeStar® Max DNA polymerase was purchased from TaKaRa. Restriction enzymes were purchased from ThermoFisher Scientific. NEBuilder® HiFi DNA Assembly Master Mix was purchased from New England Biolabs.

### Cloning, expression and purification

The genes encoding for the linear and cyclic *Is*PETase and DuraPETase variants (Life Technologies Ltd) (Table S9–10) were cloned into pET-28a(+) or pET-21b(+) expression vectors using Gibson assembly and then transformed into DH5α *E. coli*. Site directed mutagenesis was used to make the mutations listed in Table S11 with the corresponding primers. The sequences of all plasmids were confirmed by DNA Sanger sequencing performed by Eurofins Genomics.

With the exception of DuraPETase (see below), all plasmids were transformed into *E. coli* Shuffle® T7 Express cells and grown overnight at 30 °C on agar plates containing the appropriate antibiotic. The cultures were grown in Terrific Broth at 30 °C until an OD_600_ of 0.8–1.0 was reached. After induction with 0.5 mM IPTG, the culture was incubated overnight at 18 °C. DuraPETase was transformed into *E. coli* Rosetta-gami B(DE3) cells (Novagen) and recombinant expression carried out as described above using a temperature of 37 °C. All cell pellets were harvested by centrifugation (20 min, 4 °C, 4000 rpm), resuspended in lysis buffer (20 mM Tris base, pH 8.0, 2 mM MgCl_2_, 20 mM NaCl, 10% glycerol) and incubated on ice with Benzonase® Nuclease (Merck) for 15–30 min. After sonication on ice and centrifugation (30 min, 4 °C, 20,000 rpm), the supernatant was filtered and loaded onto a Ni–NTA gravity flow column previously equilibrated with wash buffer (50 mM Tris base, 120 mM NaCl, 10 mM imidazole, pH 8.0). After washing with buffers containing 10 mM and 20 mM imidazole, the bound protein was eluted with 500 mM imidazole. Further purification was carried out by size exclusion chromatography (Superdex200 26/600 or Superdex75 26/600, GE Healthcare) using a fast protein liquid chromatography system (BioRad NGC^TM^ Chromatography System QuestTM 10 Plus). The column was equilibrated with buffer (50 mM Na_2_HPO_4_, 100 mM NaCl, pH 7.5 or 8.0). After the protein sample was loaded, the column was washed and protein eluted using the same buffer. Fractions containing the desired protein were collected and concentrated using a Vivaspin® centrifugal concentrator and stored at 4 °C. Samples were analyzed by SDS-PAGE. Coomassie Brilliant Blue G (Apollo Scientific) stain was used for visualization of the protein bands, a ChemiDoc XRS+ System (BioRad) was used for imaging and Image Lab Software (Biorad) was used for image acquisition. The edges of the gel images provided in the main text have been cropped for neatness.

### Protein mass spectrometry

Protein liquid chromatography-mass spectrometry (LC–MS) was carried out by Analytical Services in the School of Chemistry at Cardiff University. The analysis was performed on a Waters Synapt G2-Si quadrupole time-of-flight mass spectrometer coupled to a Waters Acquity H-Class ultraperformance liquid chromatography (UPLC) system. The column used was a Waters Acquity UPLC Protein C4 BEH column (300 Å, 1.7 µm, 2.1 by 100 mm), held at 60 °C. A gradient of H_2_O containing 0.1% CHO_2_H and acetonitrile containing 0.1% CHO_2_H was employed. Data was collected in positive electron spray ionization mode and analyzed using Waters MassLynx software version 4.1. Maximum entropy 1 software was used to generate deconvoluted mass spectra (Figs. S17–19).

### Circular dichroism and melting temperature

CD measurements were performed on a Chirascan^TM^ CD spectrophotometer (Applied Photophysics) with a temperature control unit. Filtered and degassed buffer (50 mM Na_2_HPO_4_, pH 8.0, 100 mM NaCl) was used for all measurements. Protein samples were diluted to around 0.1 mg/mL and concentration confirmed using absorbance at 280 nm (NanoDrop^TM^ spectrophotometer). CD spectra were recorded at 20 °C, between 200 and 300 nm (in 1 nm steps), using a quartz cuvette (Hellma) with a path length of 1 mm. To follow protein thermal denaturation, the signal at 222 nm was monitored as the temperature of the sample was increased from 10 to 90 °C (in 2 °C steps). The mid-point of protein unfolding (T_m_) was then determined by the fitting of a dose–response equation. All CD data analyses were carried out using Origin2019b (OriginLab Corporation).

### Trypsin digest

A stock solution of pancreatic bovine trypsin (40 µM) (Melford) was made by dissolving 1 mg in 20 mM CaCl_2_ and 1 mM HCl, this was then diluted with buffer (50 mM Na_2_HPO_4_, pH 8.0, 100 mM NaCl) to 4 µM. The digestions of the *Is*PETase variants (10 µM) were carried out at 30, 40 and 50 °C using a trypsin-to-*Is*PETase molar ratio of 1:100. The progress of the digestion was monitored by taking SDS-PAGE samples at time intervals up to 4 h. Reactions were quenched upon addition of SDS-PAGE loading buffer followed by the addition of 8 M urea (final concentration of 5 M). Samples were analyzed by SDS-PAGE.

### *p*-NPA hydrolysis kinetics

The production of *p*-NP from the hydrolysis of *p*-NPA (Acros) was followed spectrophotometrically using a UV-2600 UV–Vis spectrophotometer (Shimadzu) by monitoring the increase in absorbance at 405 nm (A_405_) for 300 s. All measurements were carried out in 50 mM Na_2_HPO_4_, pH 7.5, 100 mM buffer at 30 °C. *p*-NPA solutions (2.5–100 mM) were prepared in DMSO from a 250 mM stock solution. Buffer was pre-incubated at 30 °C in quartz cuvettes (Hellma) for 5–10 min before the addition of *p*-NPA (1-in-20 dilution), giving final substrate concentrations of 0–5.0 mM (5% v/v DMSO). To initiate the enzymatic reaction, enzyme stock solution was added to the substrate and buffer solution, giving an enzyme concentration of 5 (*Is*PETase-Dimer and -Cat) or 10 nM (*Is*PETase-WT and -Spy). For non-enzymatic hydrolysis reactions, enzyme volume was replaced by buffer. Each measurement was repeated in triplicate. After conversion of A_405_ values into concentration of *p*-NP (ε_405_ = 18,400 M^−1^ cm^−1^), the rate of non-enzymatic hydrolysis was subtracted and the rate of enzymatic hydrolysis was calculated. Enzyme kinetics were analyzed using the Michaelis–Menten model. Data was analyzed using Origin2019b (OriginLab Corporation).

### Residual activity

For measuring residual activity, 200 µL of enzyme (250 µM of *Is*PETase-WT and -Spy, 125 µM of *Is*PETase-Dimer and -Cat) was heated at 50 °C in a PCR thermal cycler (TC-512, Techne) for 5 min–12 h, followed by incubation at 4 °C for 10 min and centrifugation. 100 µL of the supernatant was transferred into an Eppendorf tube and diluted to the appropriate concentration using buffer. Residual activity was determined using the hydrolysis of *p*-NPA, as described above. For each sample, the enzyme kinetics were analyzed as previously described using the Michaelis–Menten model. The percentage residual activity was calculated using Eq.(1), where *t* is the time the sample was heated at 50 °C.

1 $$\% \,Residual\,Activity = \frac{{\left[ {k_{cat} /K_{m} } \right]_{t = x} }}{{\left[ {k_{cat} /K_{m} } \right]_{t = 0} }} \times 100$$

### PET hydrolysis

The PET degradation activities of the *Is*PETase variants, were investigated according to a modified procedure reported by Arnling Bååth et al.^49^. Absorbance spectra were recorded between 200 and 500 nm using a UV-2600 UV–Vis spectrophotometer (Shimadzu) and quartz cuvettes (Hellma). Semi-crystalline PET powder (Goodfellow) was suspended in 50 mM Na_2_HPO_4_ (pH 8.0) and 100 mM NaCl buffer. Reactions were performed in triplicate on a 1 mL scale in Eppendorf tubes, with a PET concentration of 7.5 mg/mL. Enzymes were used in concentrations of 0.1 (*Is*PETase-WT or -Spy) or 0.05 µM (*Is*PETase-Dimer or -Cat). For PET only and enzyme only control samples, the appropriate volume of buffer was used to replace the enzyme or the PET suspension, respectively. Samples were incubated in a ThermoMixer^TM^ (Eppendorf) at the required temperature (30, 40, or 50 °C) with shaking (550 or 1100 rpm) or without shaking (0 rpm). At selected time intervals, samples were quenched by centrifugation (10 min, 4 °C) and the supernatant analyzed using absorbance at 240 nm (A_240_). Measurements were made in triplicate and the A_240_ averaged. The A_240_ measured for the enzyme only control samples were subtracted and converted into the concentration soluble degradation products (ε_240_ = 13,800 M^−1^ cm^−1^).

For the DuraPETase variants, PET degradation experiments and analyses were carried out as described above. Incubation temperatures of 30, 50 and 70 °C were used with shaking speeds of 0 and 1100 rpm.

## Supplementary Information

Supplementary Information.

## Data Availability

The gene sequences for the cyclised *Is*PETase and DuraPETase variants have been verified by Sanger sequencing through commercial vendor Eurofins and will be available in GenBank under the accession numbers OP594446-OP594453.
