# Supplementary material for: Investigating the effects of cyclic topology on the performance of a plastic degrading enzyme for polyethylene terephthalate degradation
Source: Sci Rep. 2023 Jan 23;13:1267. doi: 10.1038/s41598-023-27780-4 (PMC9870871; doi:10.1038/s41598-023-27780-4)

**Supporting Information**

**Investigating the effects of cyclic topology on the performance of a plastic degrading enzyme for polyethylene terephthalate degradation**

Heather C. Hayes^1,2^, Louis Y.P. Luk*^1,2^

^1^ School of Chemistry, Cardiff University Main Building, Park Pl, Cardiff CF10 3AT

^2^ Cardiff Catalysis Institute, Cardiff University Main Building, Park Pl, Cardiff CF10 3AT

Correspondence: lukly@cardiff.ac.uk

**
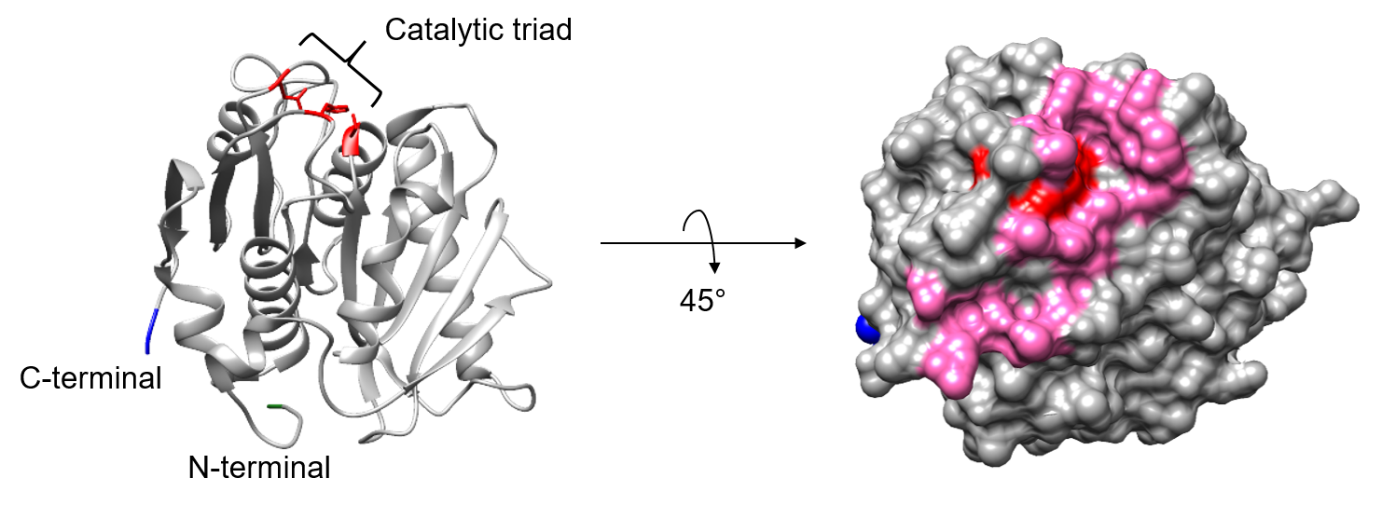
**

**Figure S1.** Crystal structure of *Is*PETase (PDB 5XJH). The catalytic triad (S160-H237-D206) is shown in red, the N-terminal is shown in green, the C-terminal is shown in blue and the amino acid residues composing the L-shaped substrate binding site (as reported by Joo et al, 2018) are shown in pink.

**
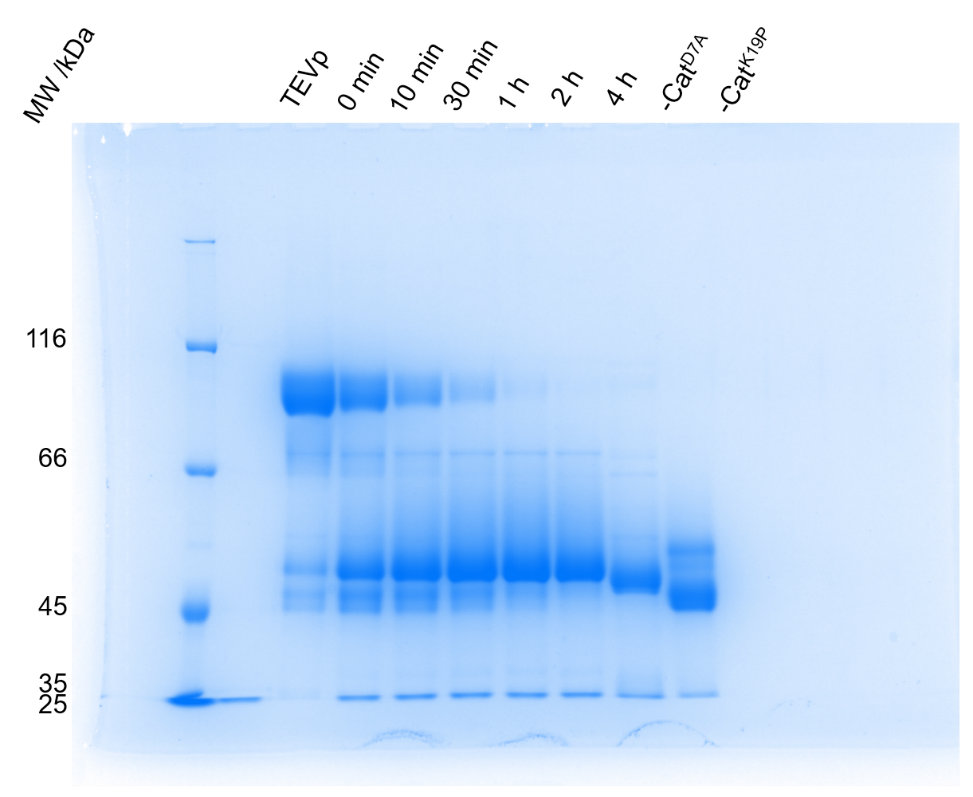
**

**Figure S2.** Original SDS-PAGE of the TEV protease (TEVp) digestion of *Is*PETase-Cat.


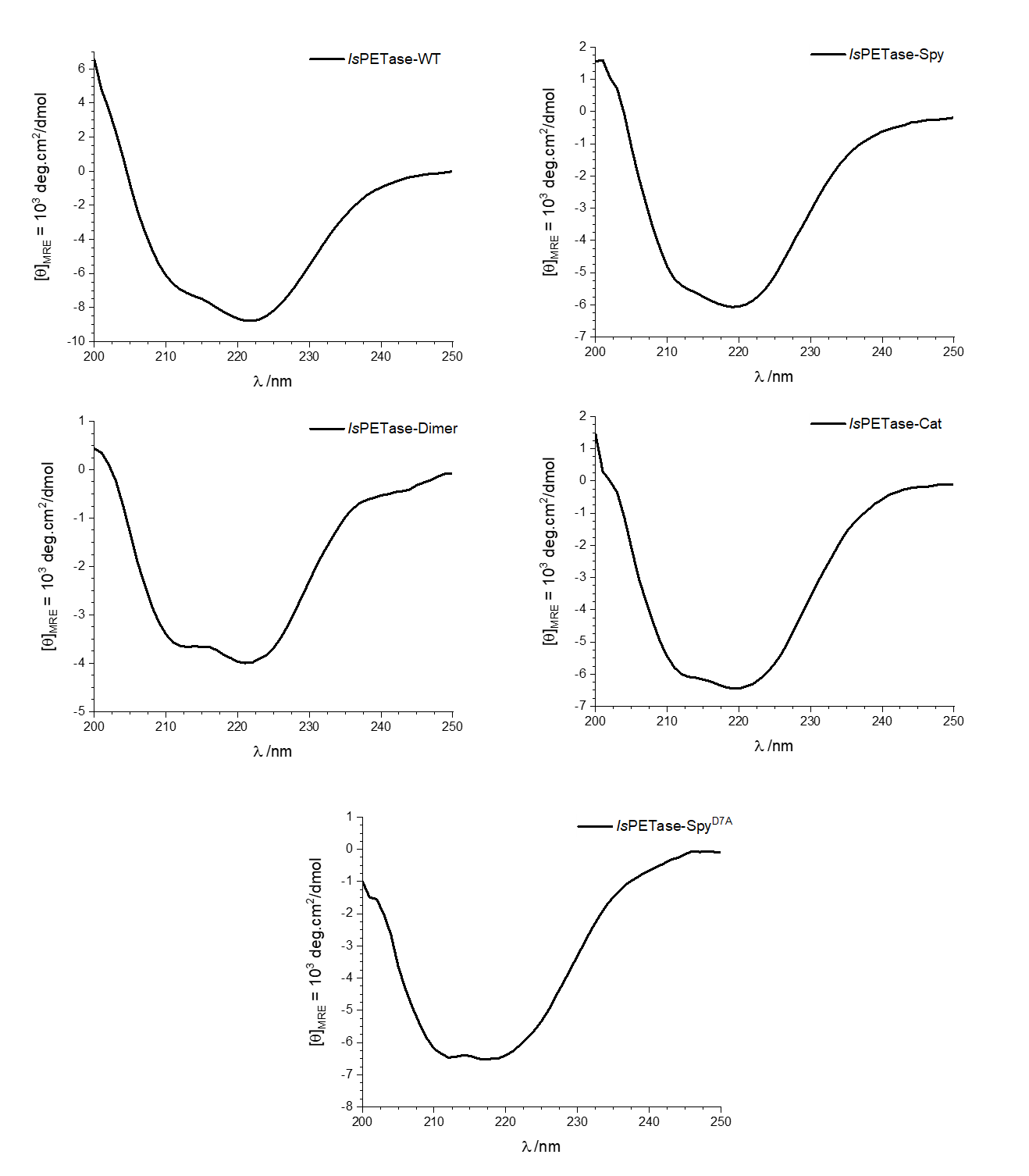


**Figure S3.** CD spectra of the linear and cyclic *Is*PETase variants at 20 °C, in 50 mM Na_2_HPO_4_ (pH 7.5) and 100 mM NaCl buffer.


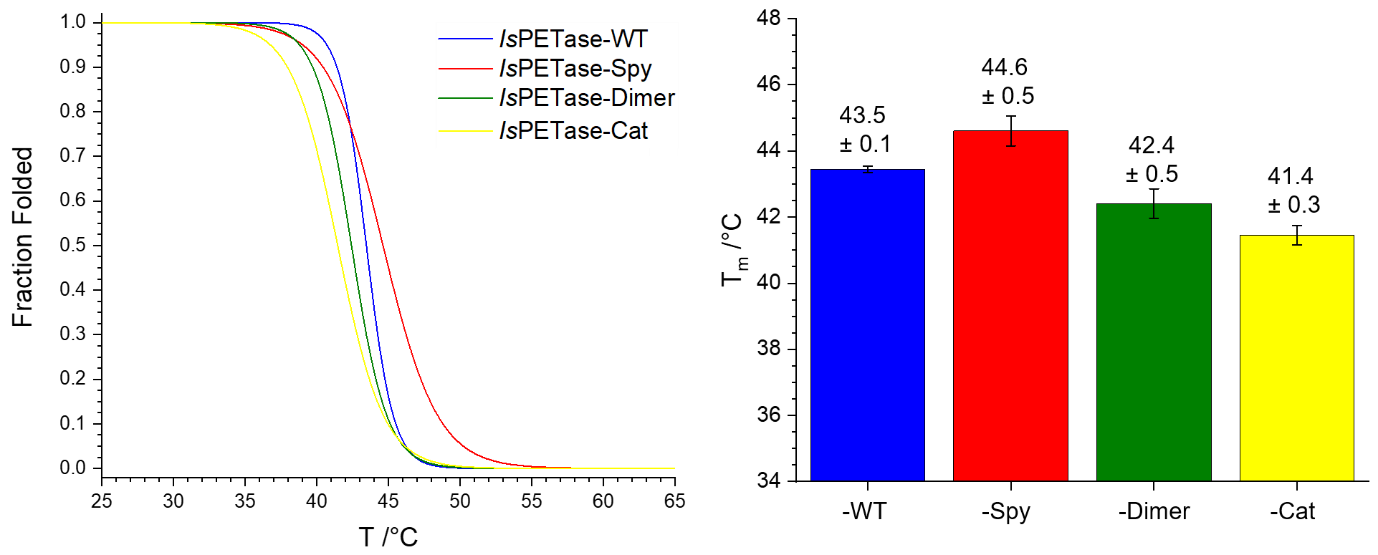


**Figure S4.** (Left) Thermal denaturation curves of *Is*PETase-WT, -Spy and –Dimer and –Cat. (Right) Bar chart comparing T_m_. Error bars represent the 95% confidence interval.

**
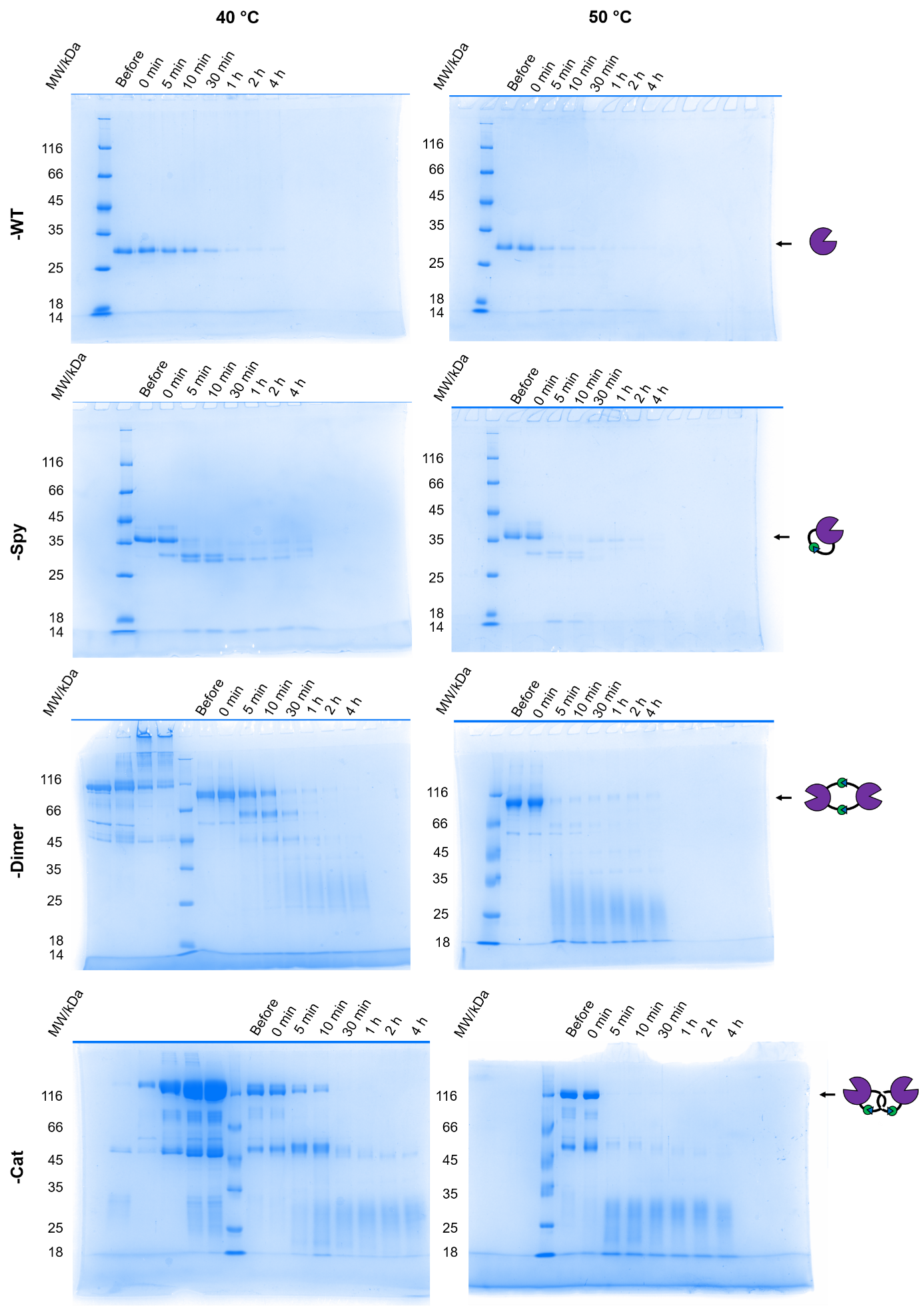
**

**Figure S5.** SDS-PAGE analysis of trypsin digest of the *Is*PETase variants at 40 and 50 °C. 50 mM Na_2_HPO_4_ (pH 8.0) and 100 mM NaCl buffer was used with a 1:100 molar ratio of trypsin-to-*Is*PETase.


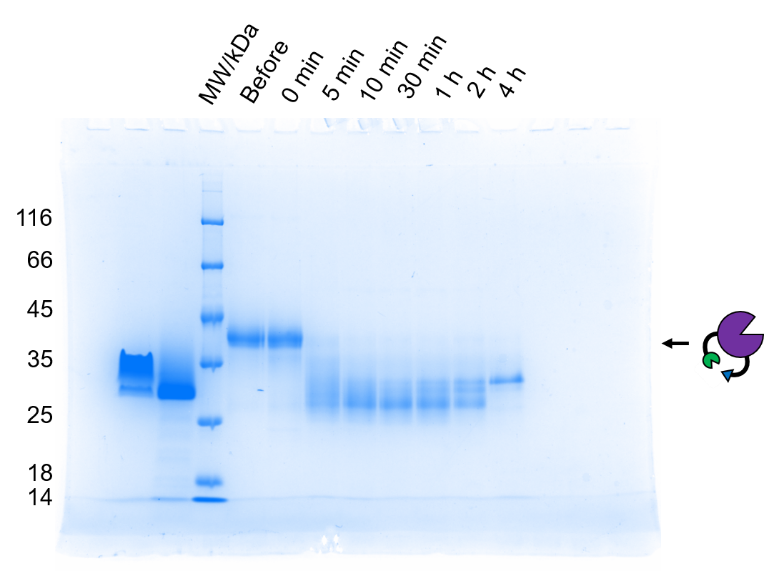


**Figure S6.** SDS-PAGE analysis of trypsin digest of the *Is*PETase-Spy^D7A^ linear control variants at 30 °C. 50 mM Na_2_HPO_4_ (pH 8.0) and 100 mM NaCl buffer was used with a 1:100 molar ratio of trypsin-to-*Is*PETase.


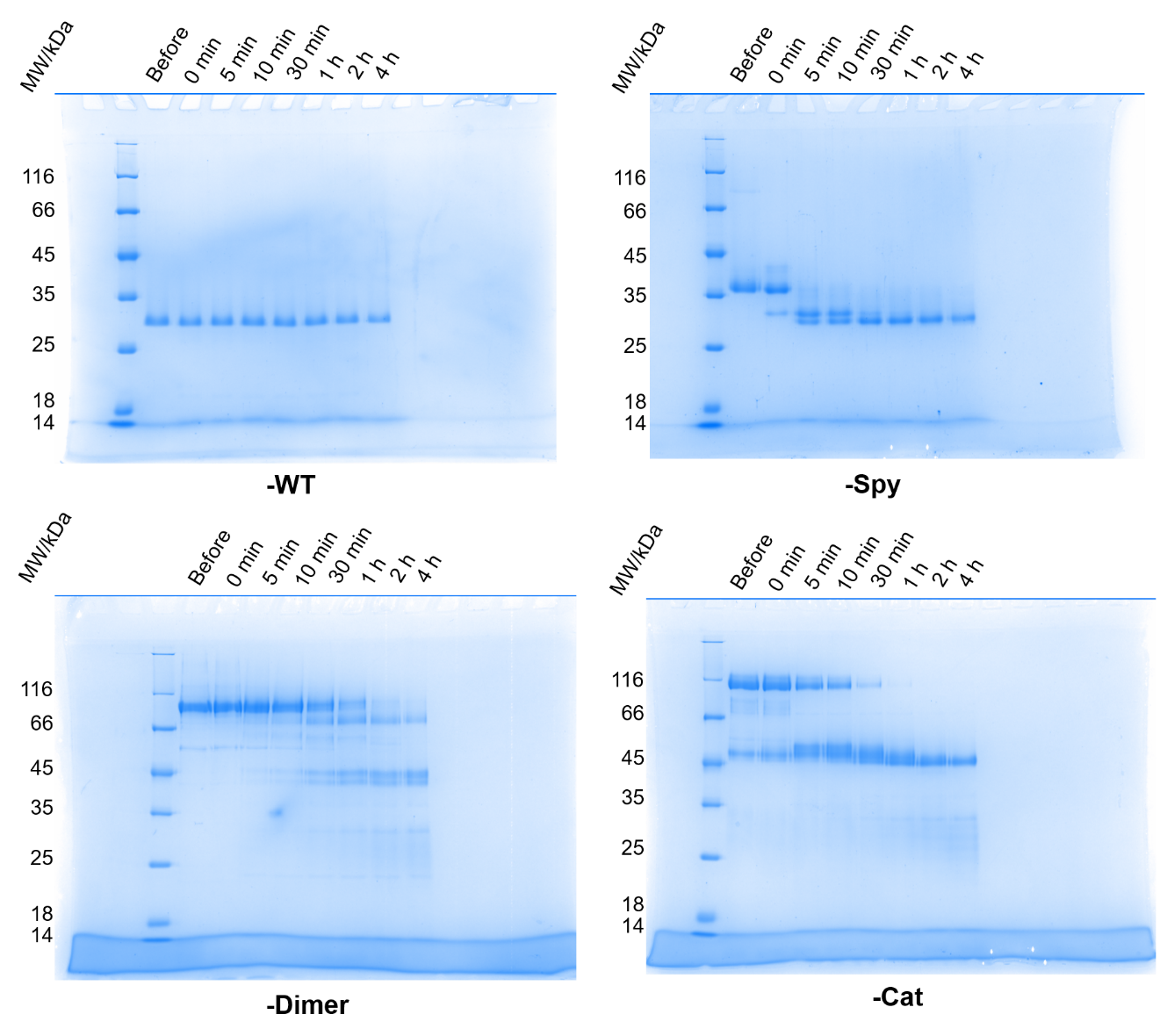


**Figure S7.** Original SDS-PAGE analysis of trypsin digest of the *Is*PETase variants at 30 °C. 50 mM Na_2_HPO_4_ (pH 8.0) and 100 mM NaCl buffer was used with a 1:100 molar ratio of trypsin-to-*Is*PETase.


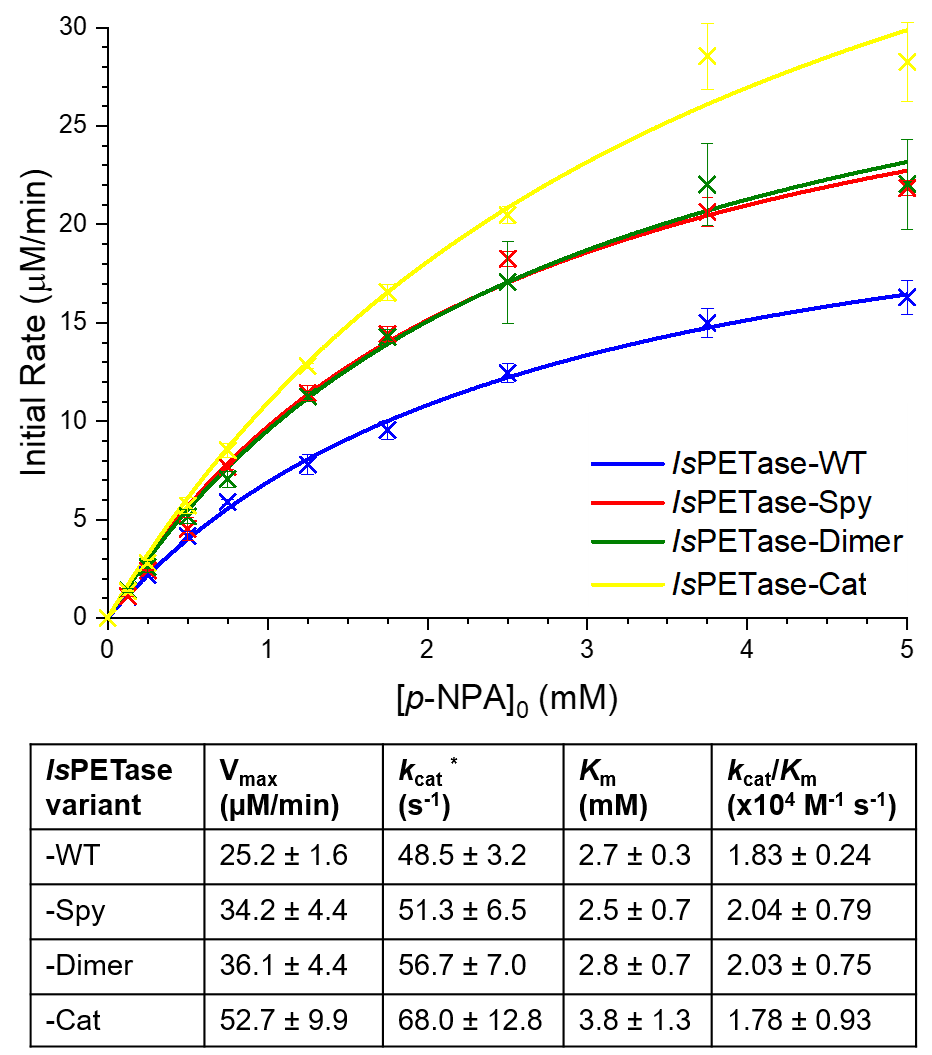


**Figure S8.** *Is*PETase-WT, -Spy, -Dimer and –Cat kinetics for the hydrolysis of *p*-NPA at 30 °C and pH 7.5, calculated using the Michaelis-Menten model. Error bars represent the standard deviation from the mean. See also **Table S5**.


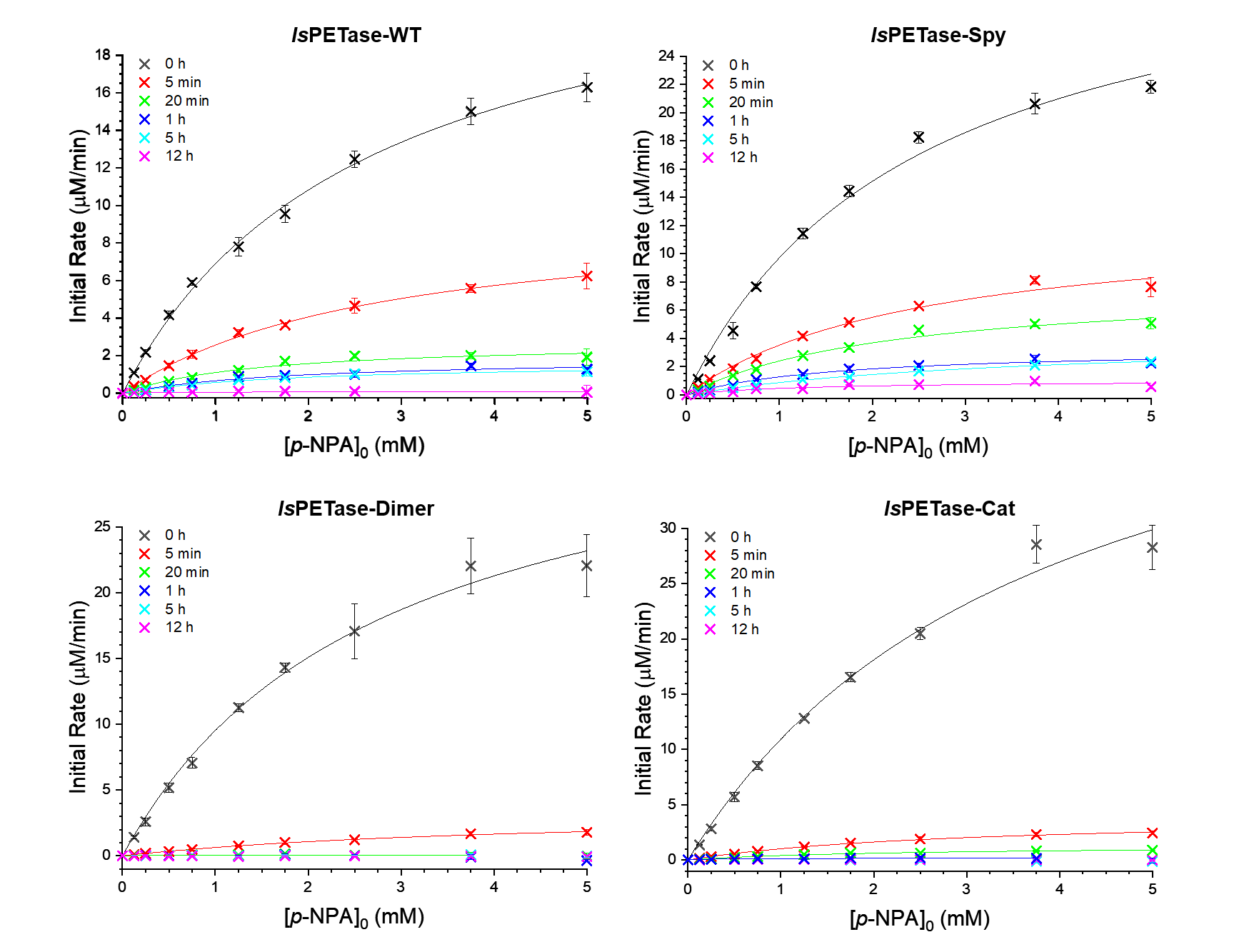


**Figure S9.** Residual activity kinetics of the *Is*PETase variants after heating for increasing lengths of time at 50 °C, measured using the hydrolysis of *p*-NPA at 30 °C and pH 7.5. Error bars represent the standard deviation from the mean. See also **Table S5**.


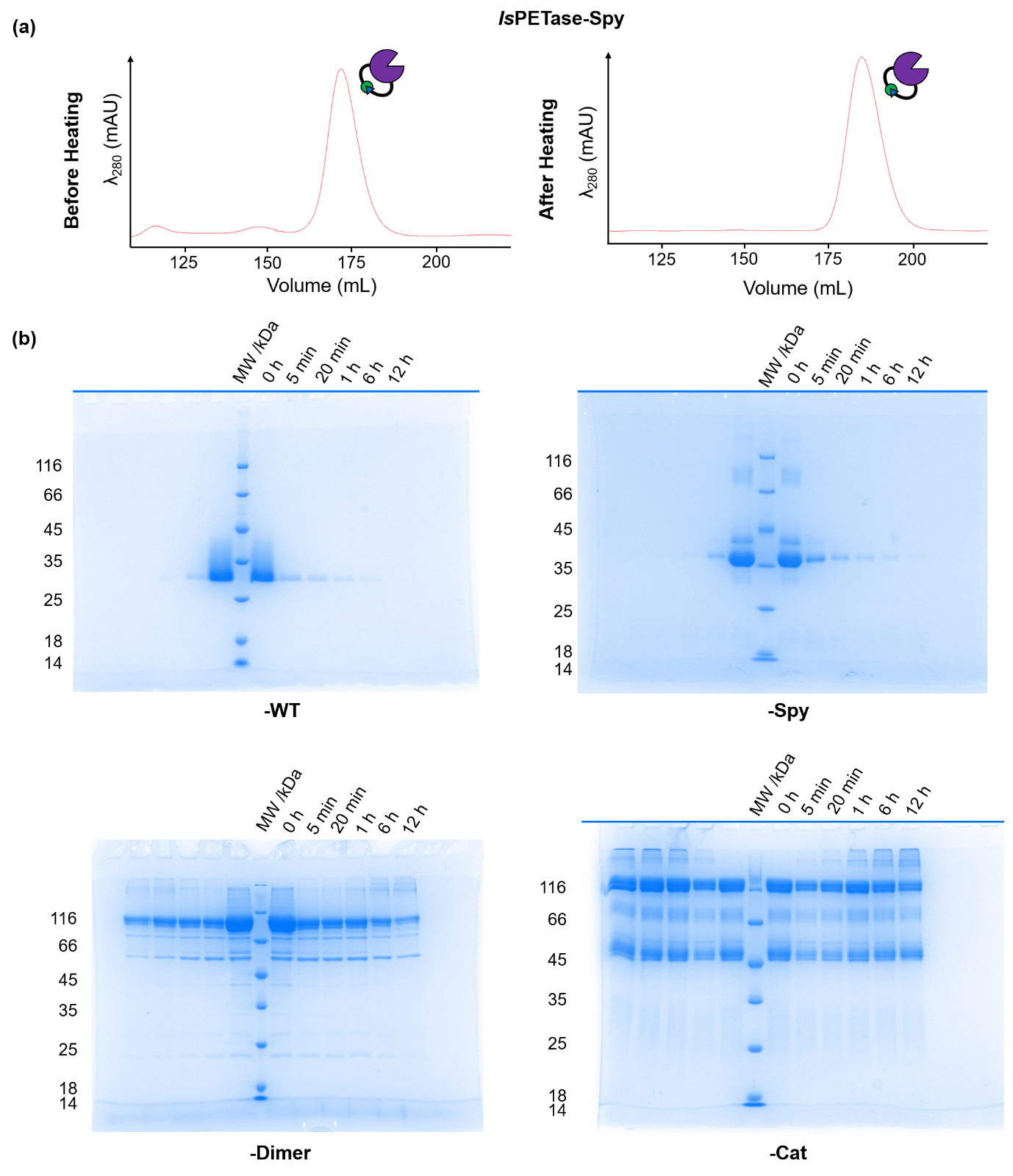


**Figure S10.** Analyses of *Is*PETase sample supernatant after heat treatment and centrifugation. (a) SEC chromatograms of *Is*PETase-Spy before and after heating at 50 °C for 5 min. (b) Original SDS-PAGE analysis of the linear and cyclic *Is*PETase variants after heating at 50 °C for increasing lengths of time.


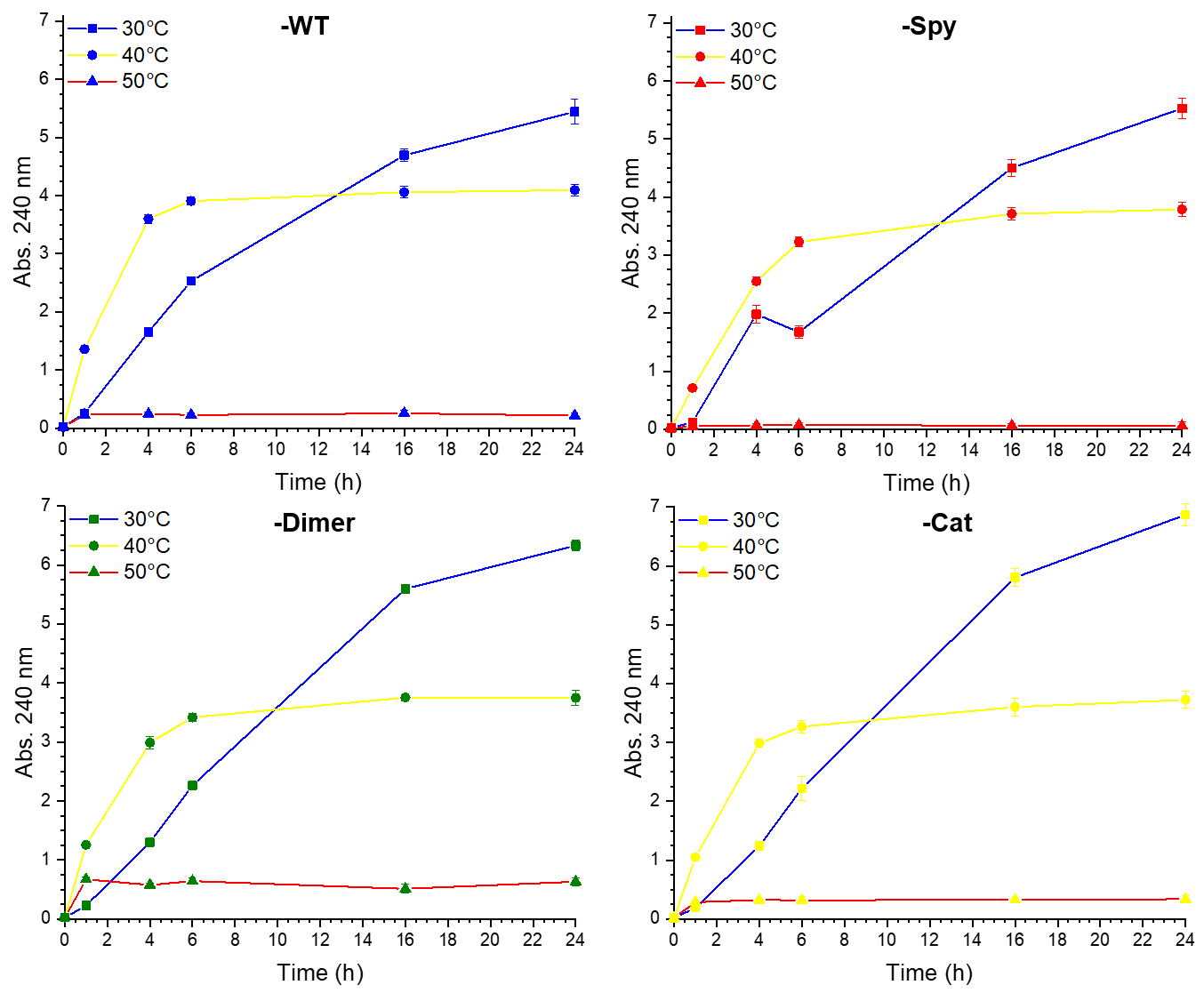


**Figure S11.** PET degrading activity of the linear and cyclic *Is*PETase variants at increasing temperatures at 0 rpm. Soluble degradation product release measured using absorbance at 240 nm (mAU) over 24 hours. Error bars represent the standard deviation from the mean. See also **Table S4**.


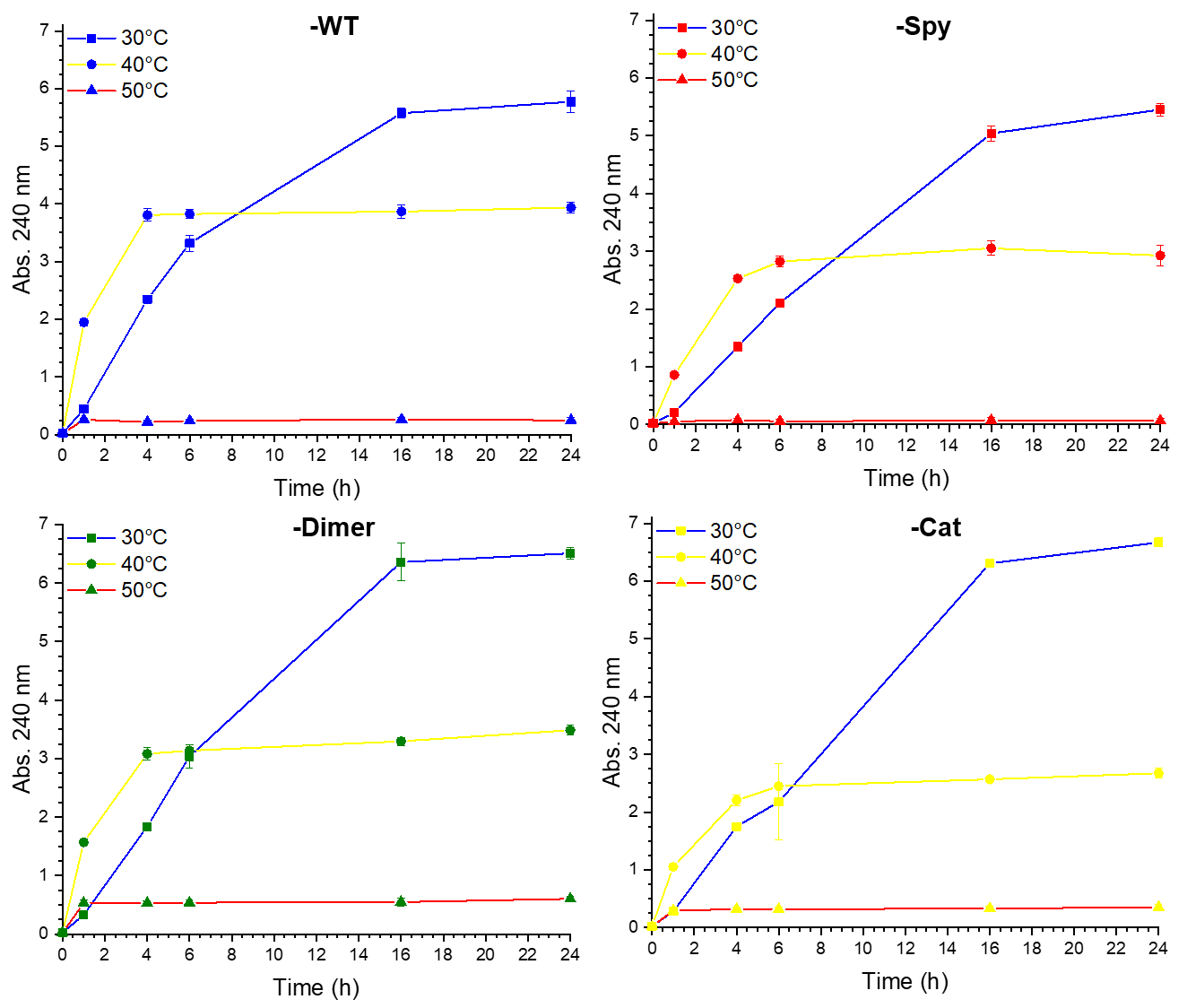


**Figure S12.** PET degrading activity of the linear and cyclic *Is*PETase variants at increasing temperatures at 550 rpm. Soluble degradation product release measured using absorbance at 240 nm (mAU) over 24 hours. Error bars represent the standard deviation from the mean. See also **Table S5**.


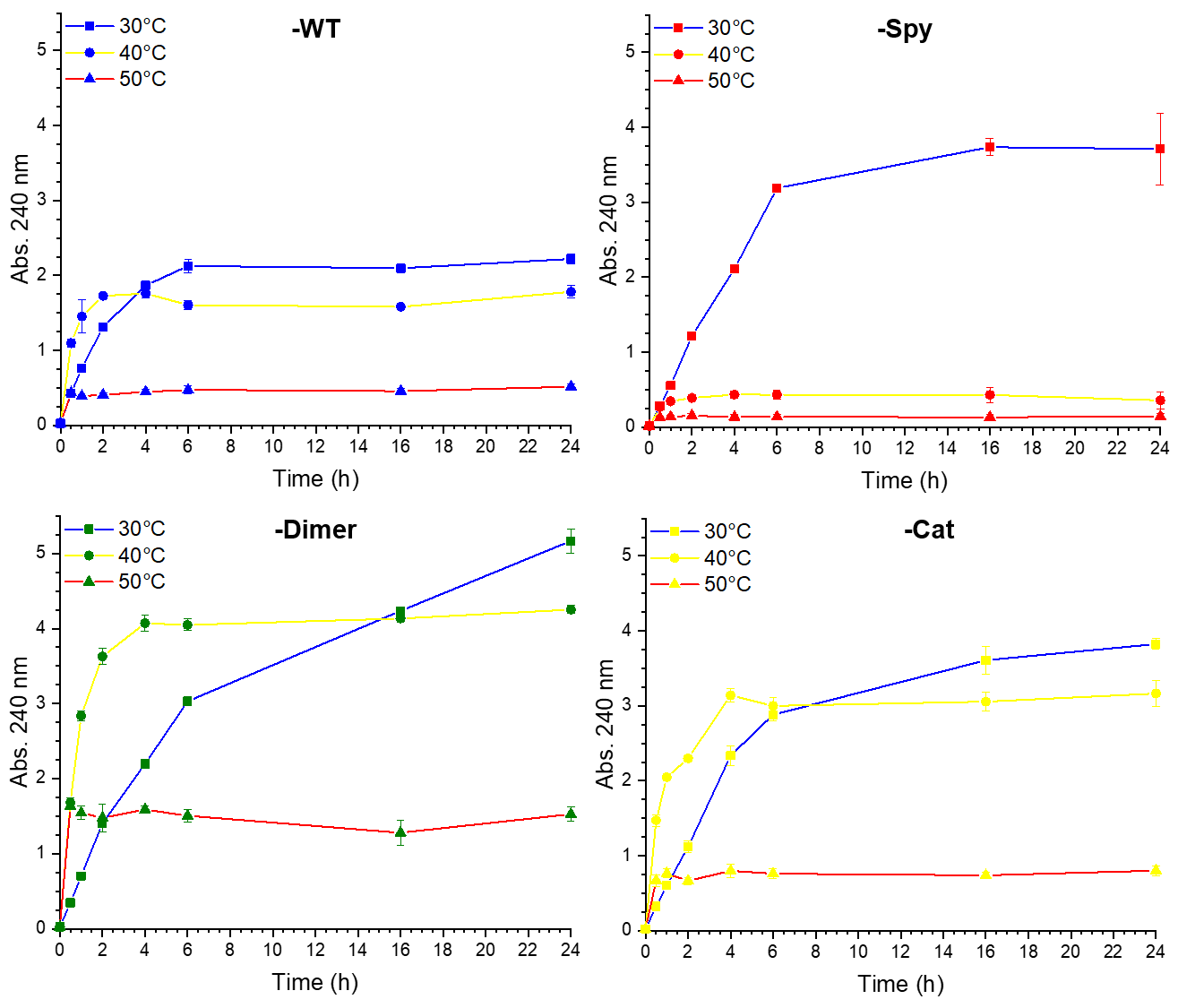


**Figure S13.** PET degrading activity of the linear and cyclic *Is*PETase variants at increasing temperatures at 1100 rpm. Soluble degradation product release measured using absorbance at 240 nm (mAU) over 24 hours. Error bars represent the standard deviation from the mean. See also **Table S6**.


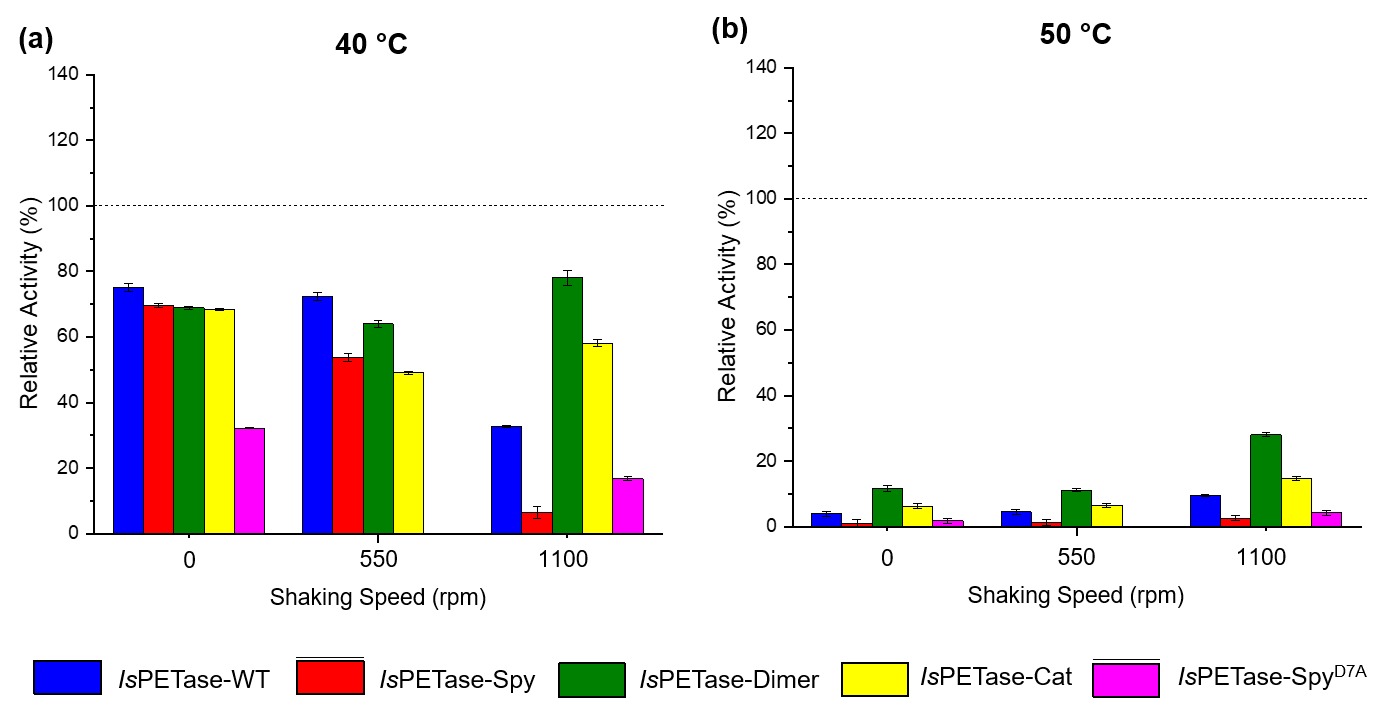


**Figure S14.** Relative PET degrading activity of the linear and cyclic *Is*PETase variants as a percentage of the concentration of soluble degradation products released by *Is*PETase-WT (i.e. 395 ± 16 µM) after incubation with 7.5 mg/mL PET for 24 hours at 30 °C, 0 rpm. (a) Incubation with PET at 40 °C. (b) Incubation with PET at 50 °C. Error bars represent the standard deviation from the mean.


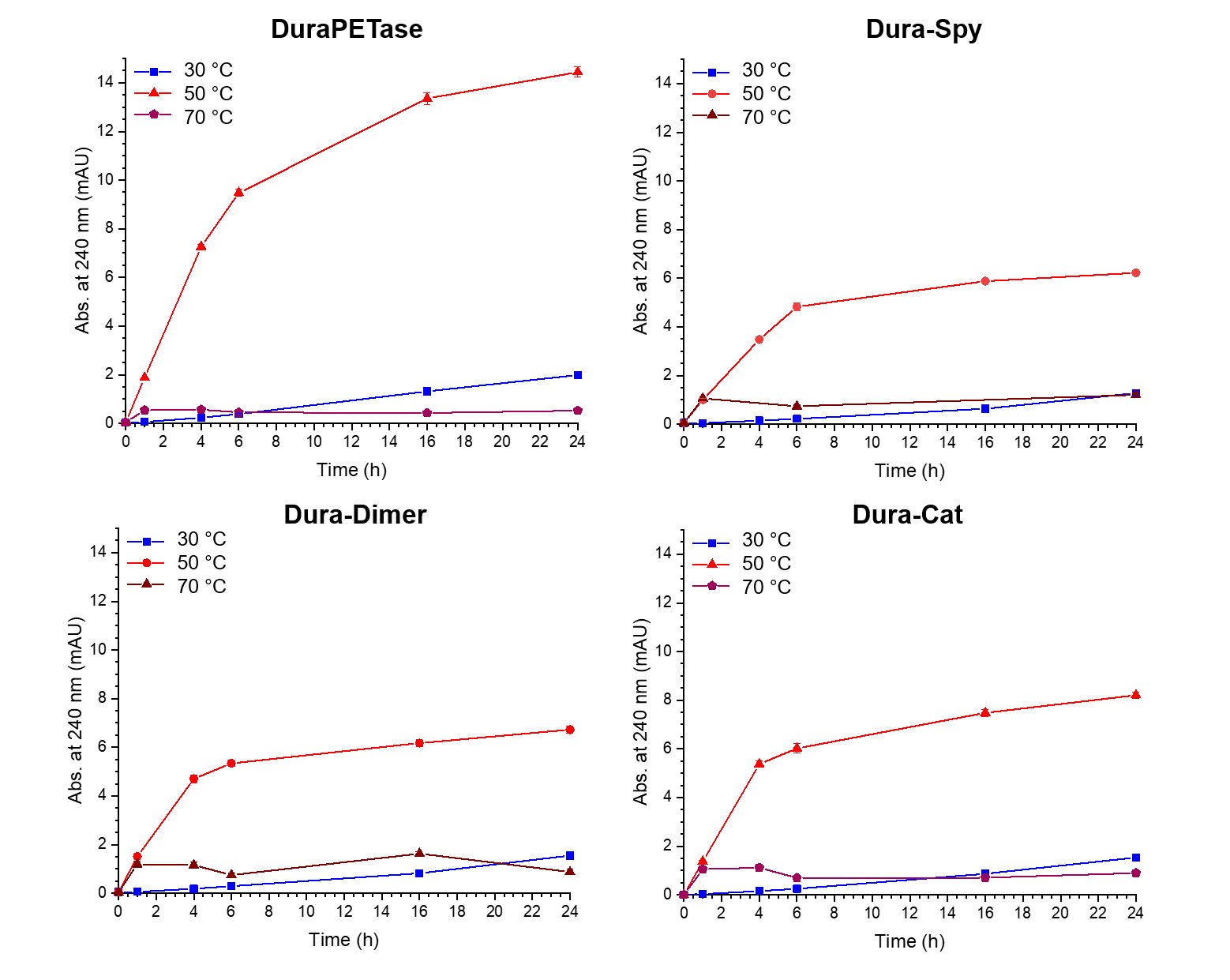


**Figure S15.** PET degrading activity of the linear and cyclic DuraPETase variants at increasing temperatures at 0 rpm. Soluble degradation product release measured using absorbance at 240 nm (mAU) over 24 hours. Error bars represent the standard deviation from the mean. See also **Table S7**.

**
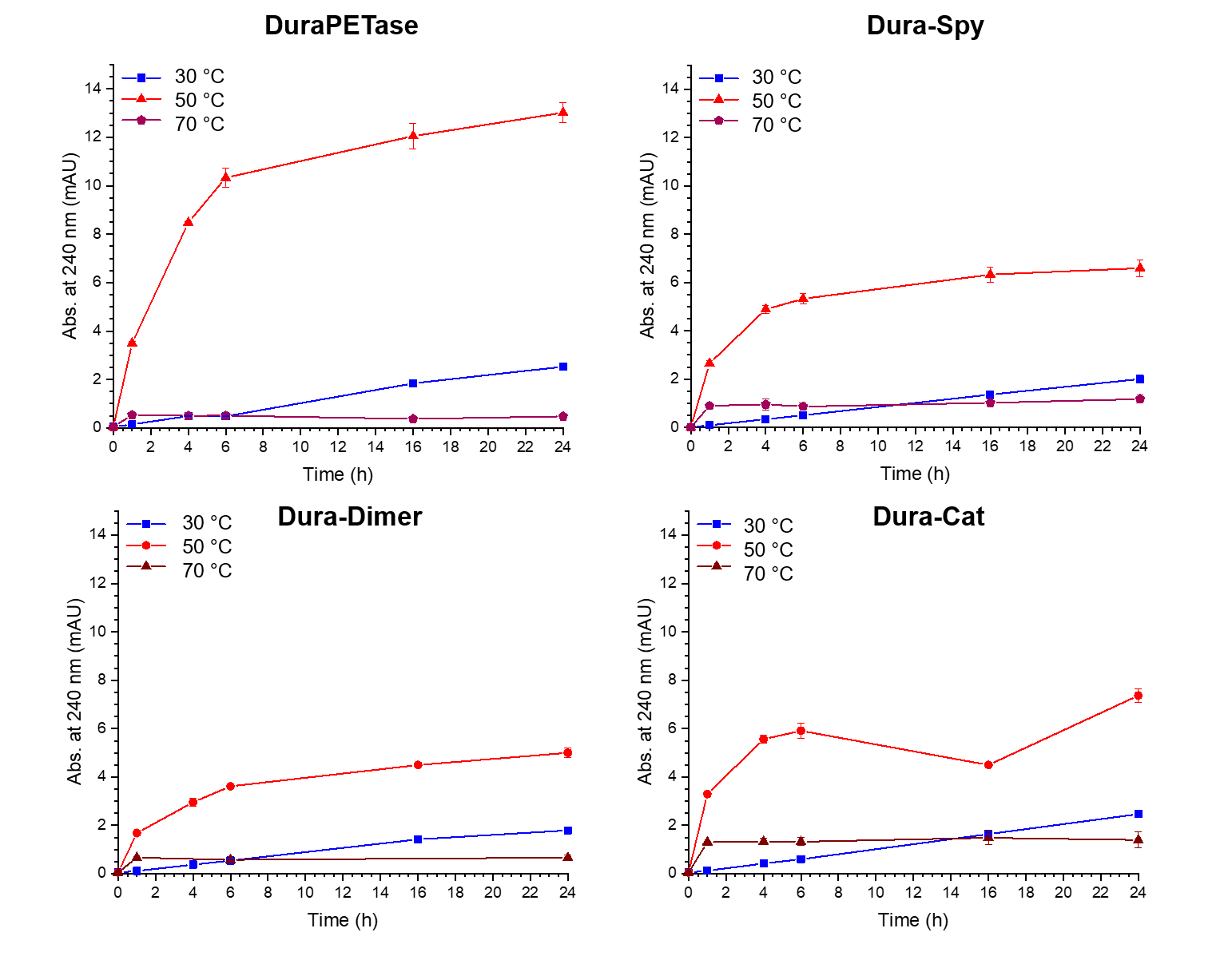
**

**Figure S16.** PET degrading activity of the linear and cyclic DuraPETase variants at increasing temperatures at 0 rpm. Soluble degradation product release measured using absorbance at 240 nm (mAU) over 24 hours. Error bars represent the standard deviation from the mean. See also **Table S8**.

**
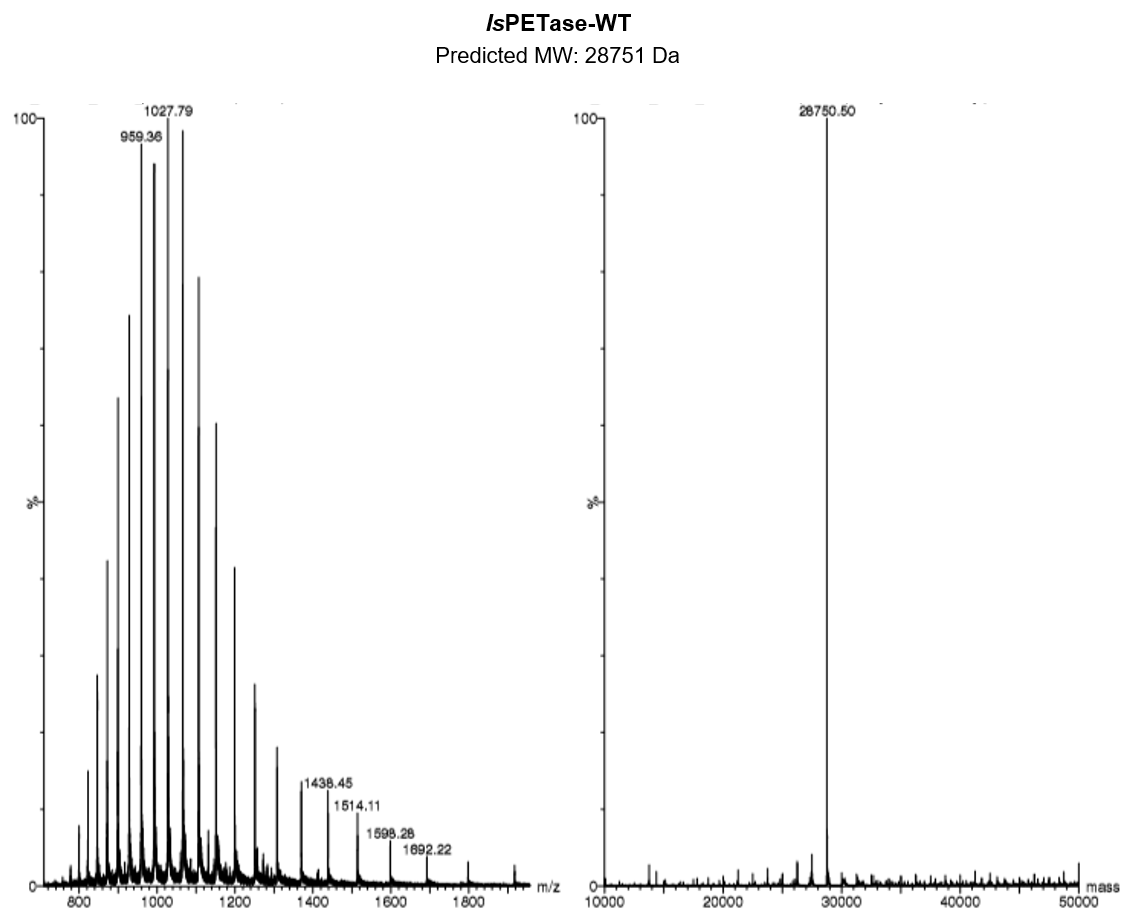
**

**Figure S17.** Mass spectra of *Is*PETase-WT. The molecular weight of the sample was predicted using the Expasy Protoparam online tool and takes into account disulfide bond formation (2H loss x2).

**
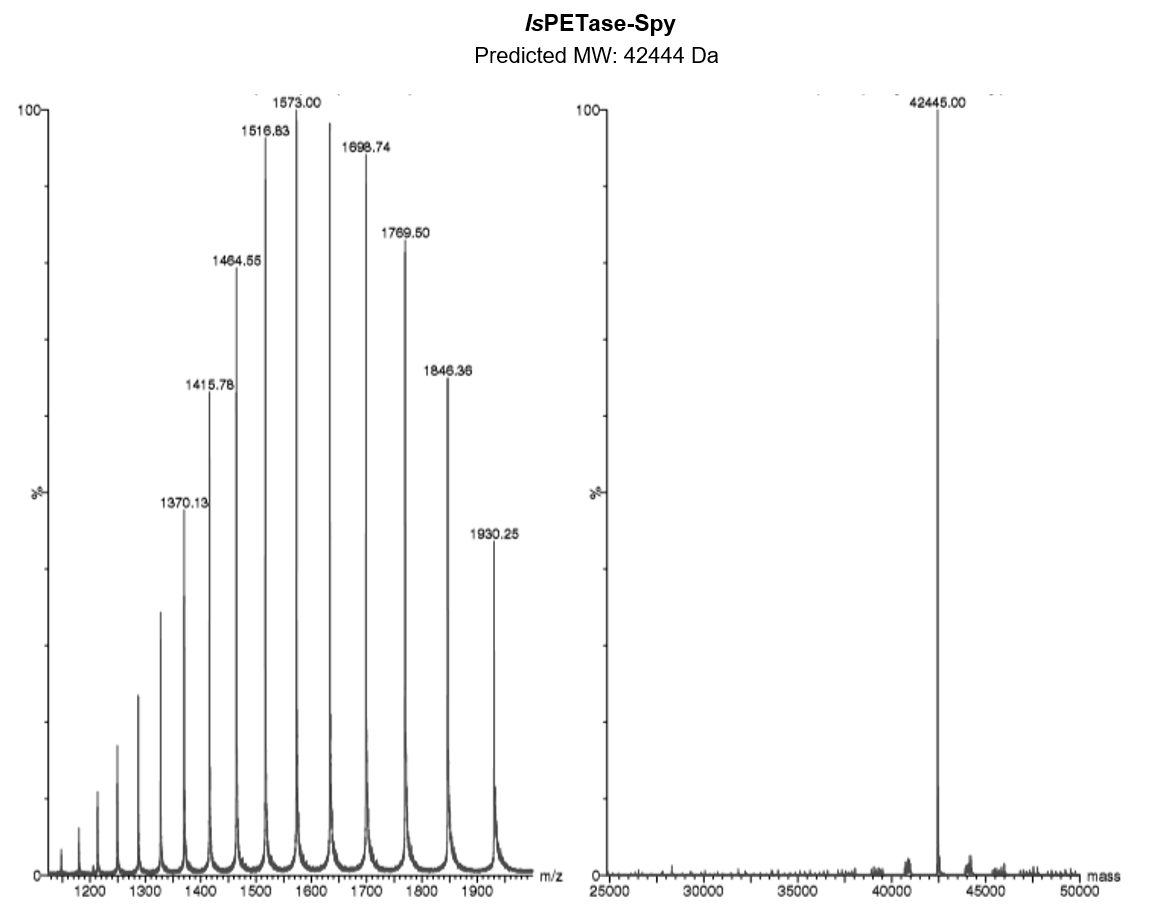
**

**Figure S18.** Mass spectra of *Is*PETase-Spy. The molecular weight of the sample was predicted using the Expasy Protoparam online tool and takes into account disulfide bond formation (2H loss x2) and the loss of one molecule of H_2_O due to isopeptide bond formation.

**
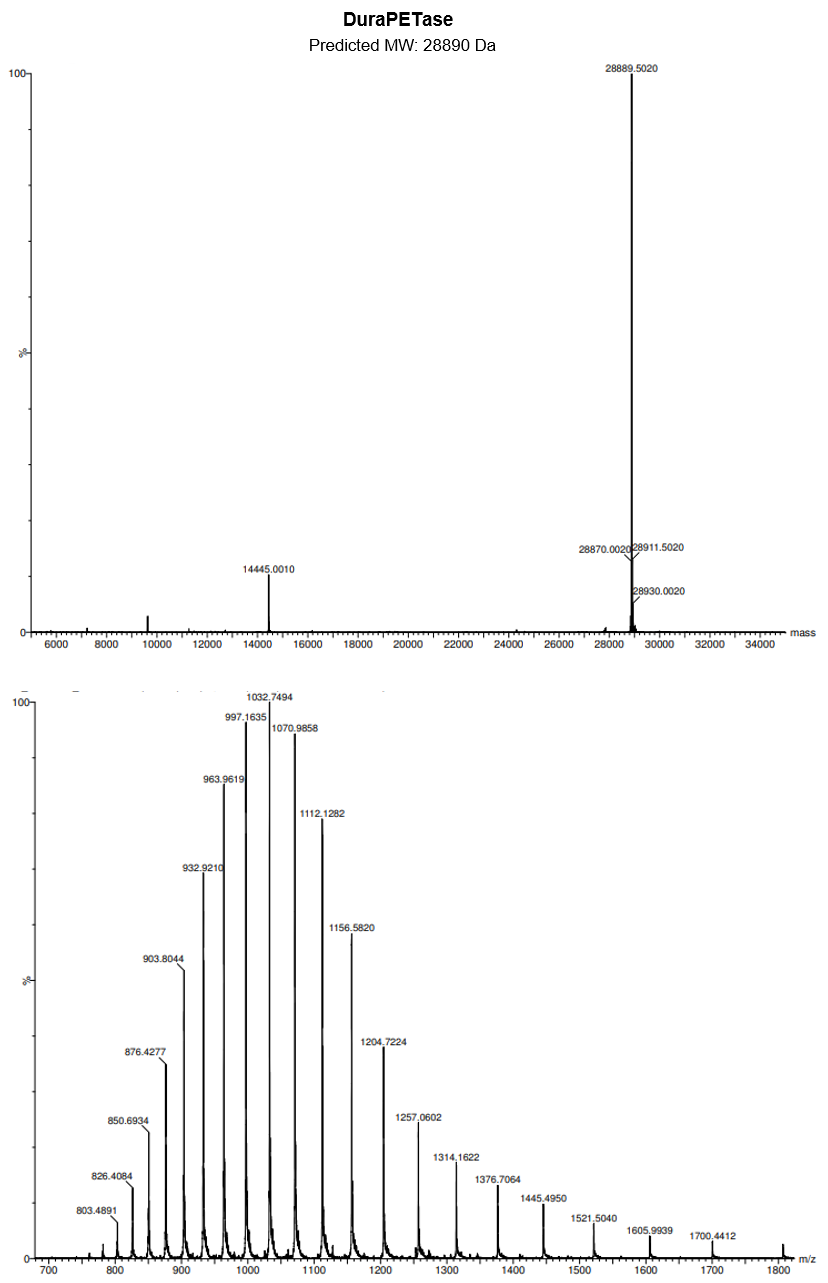
**

**Figure S19.** Mass spectra of DuraPETase. The molecular weight of the sample was predicted using the Expasy Protoparam online tool and calculations take into account disulfide bond formation (2H loss x2).

**Table S1.** Table summarizing the T_m_ measured for all *Is*PETase variants.


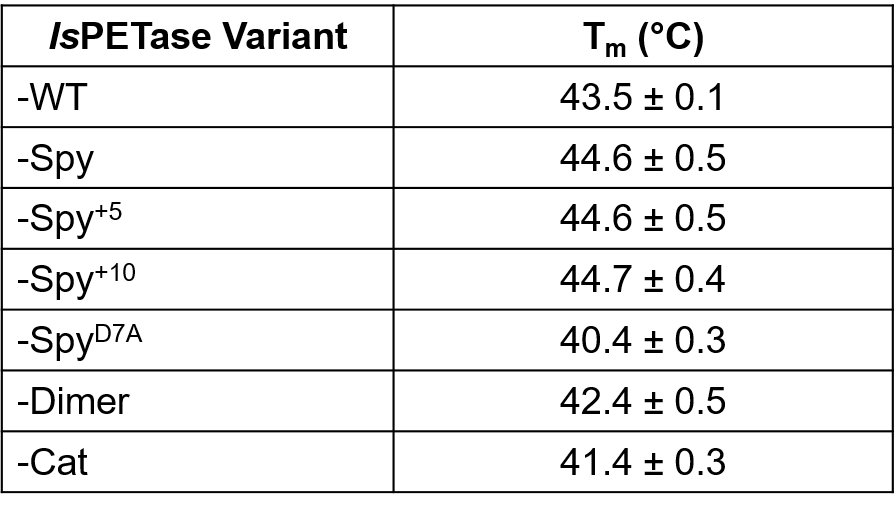


**Table S2.** Residual activity of the *Is*PETase variants after heating at 50 °C for increasing lengths of time. Kinetics were measured using the hydrolysis of *p*-NPA at 30 °C and pH 7.5, calculated using the Michaelis-Menten model. **k*_cat_ = V_max_/[E], where [E] is the concentration of active sites. Error bars represent the standard deviation from the mean. See also **Figure S8**-**9**.


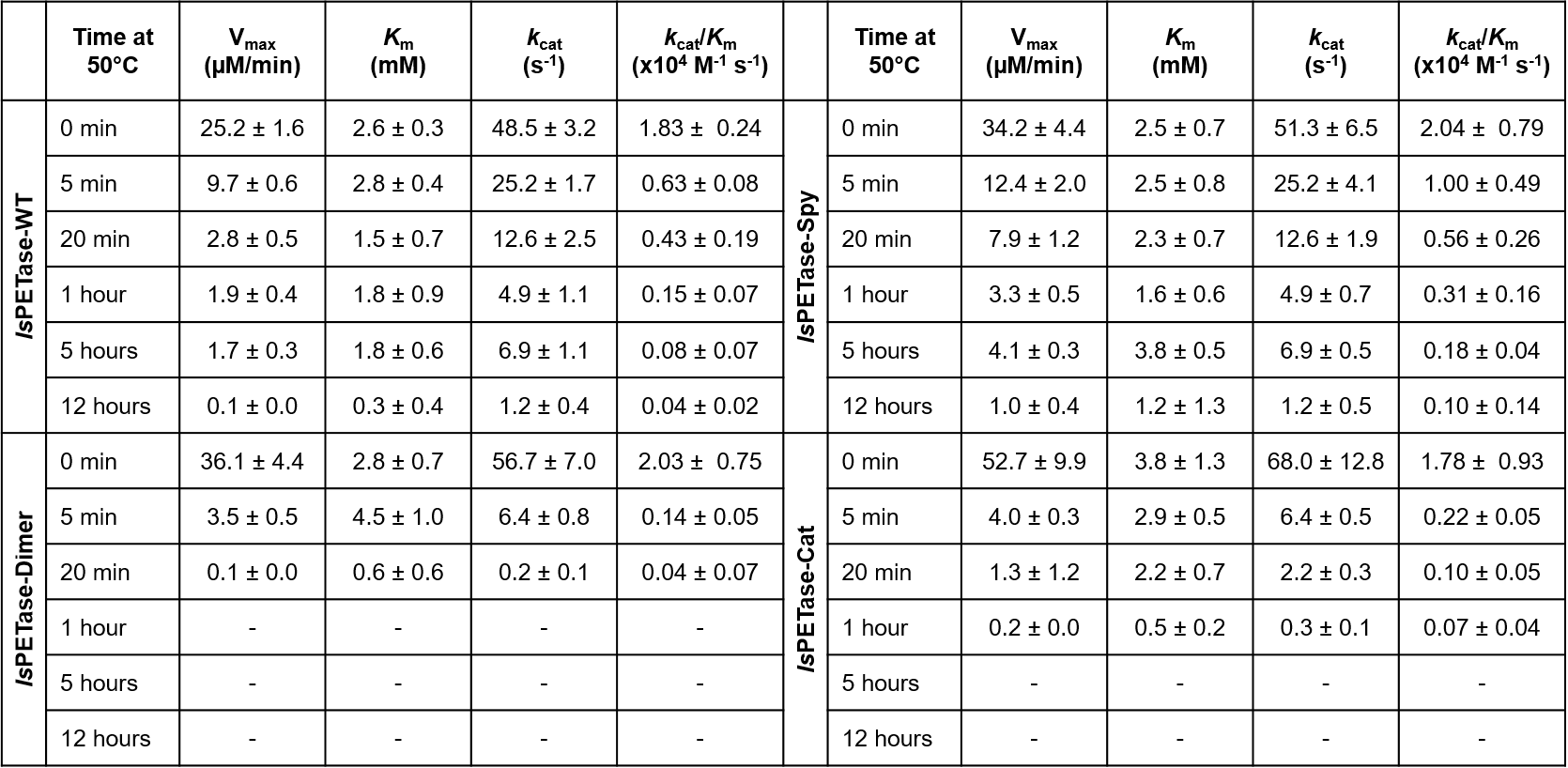


**Table S3.** Table comparing the cyclic monomer constructs of *Is*PETase created in this work (*Is*PETase-Spy) and in the patent (WO/2021/14pNP5822). Differences in the experiments carried out to assess enzyme stability are also summarized.

**
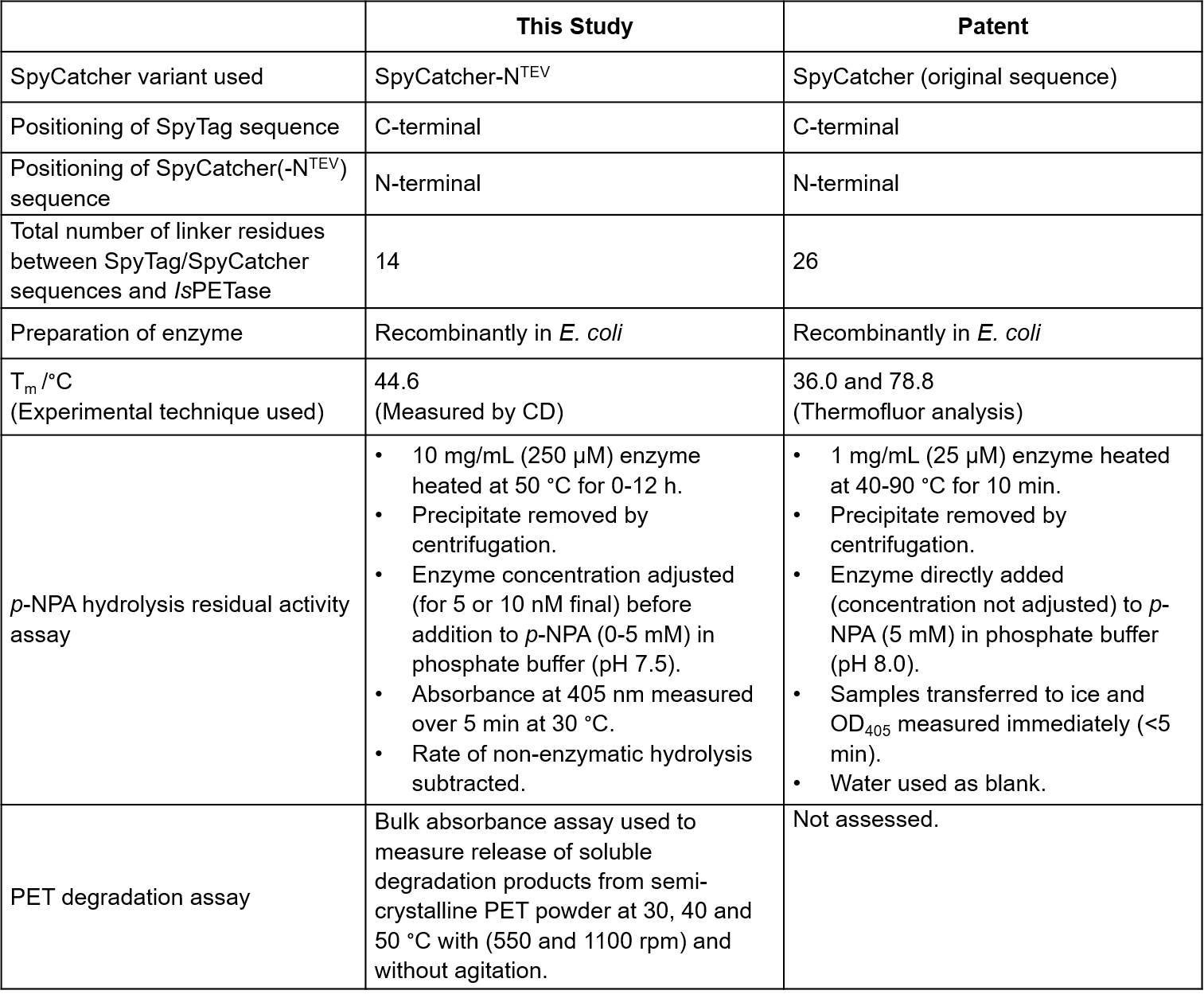
**

**Table S4.** PET degrading activities of the linear and cyclic *Is*PETase variants at increasing temperature at 0 rpm. Soluble degradation product release monitored over 24 hours using the absorbance at 240 nm (mAU).


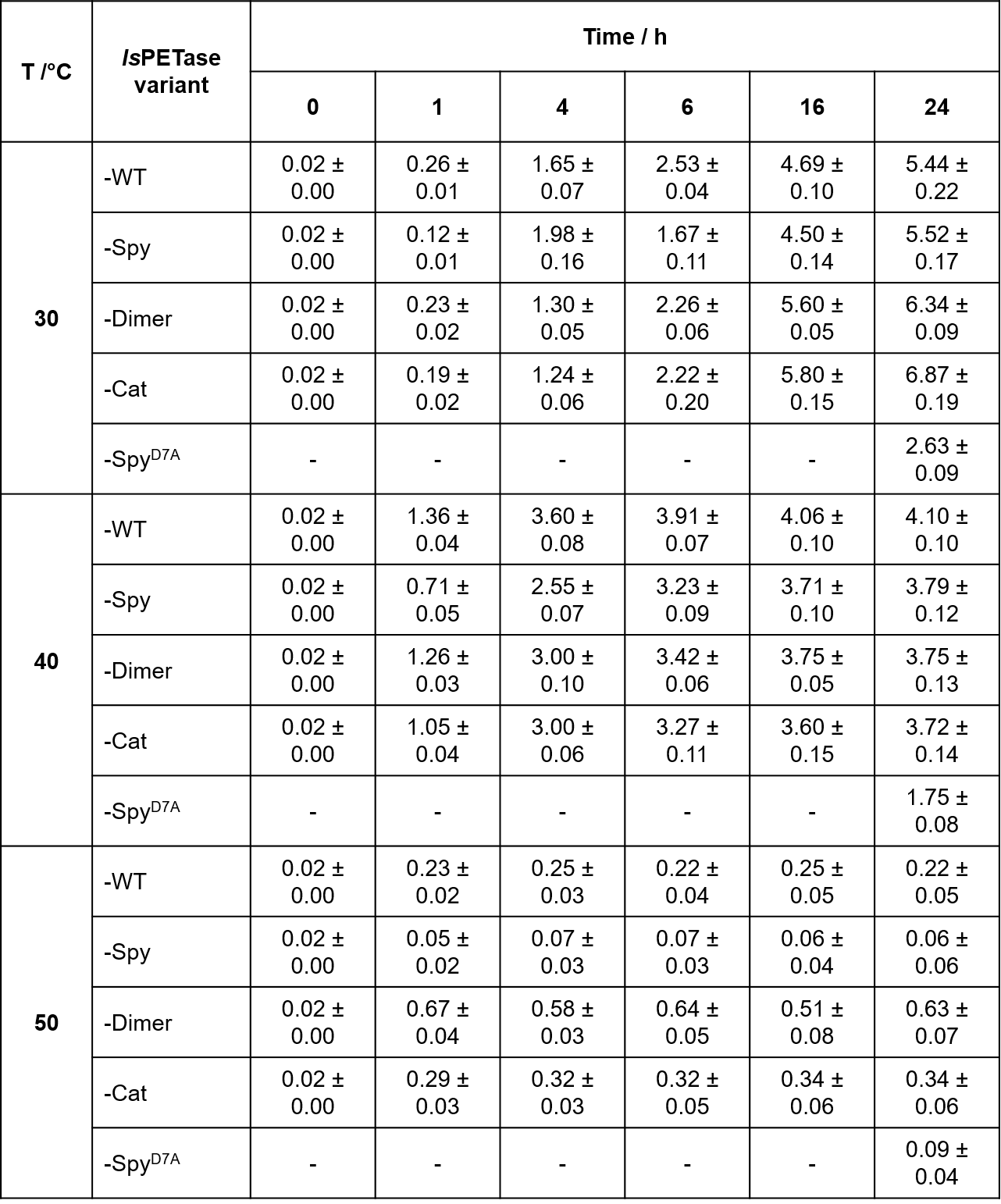


**Table S5.** PET degrading activities of the linear and cyclic *Is*PETase variants at increasing temperature at 550 rpm. Soluble degradation product release monitored over 24 hours using the absorbance at 240 nm (mAU).


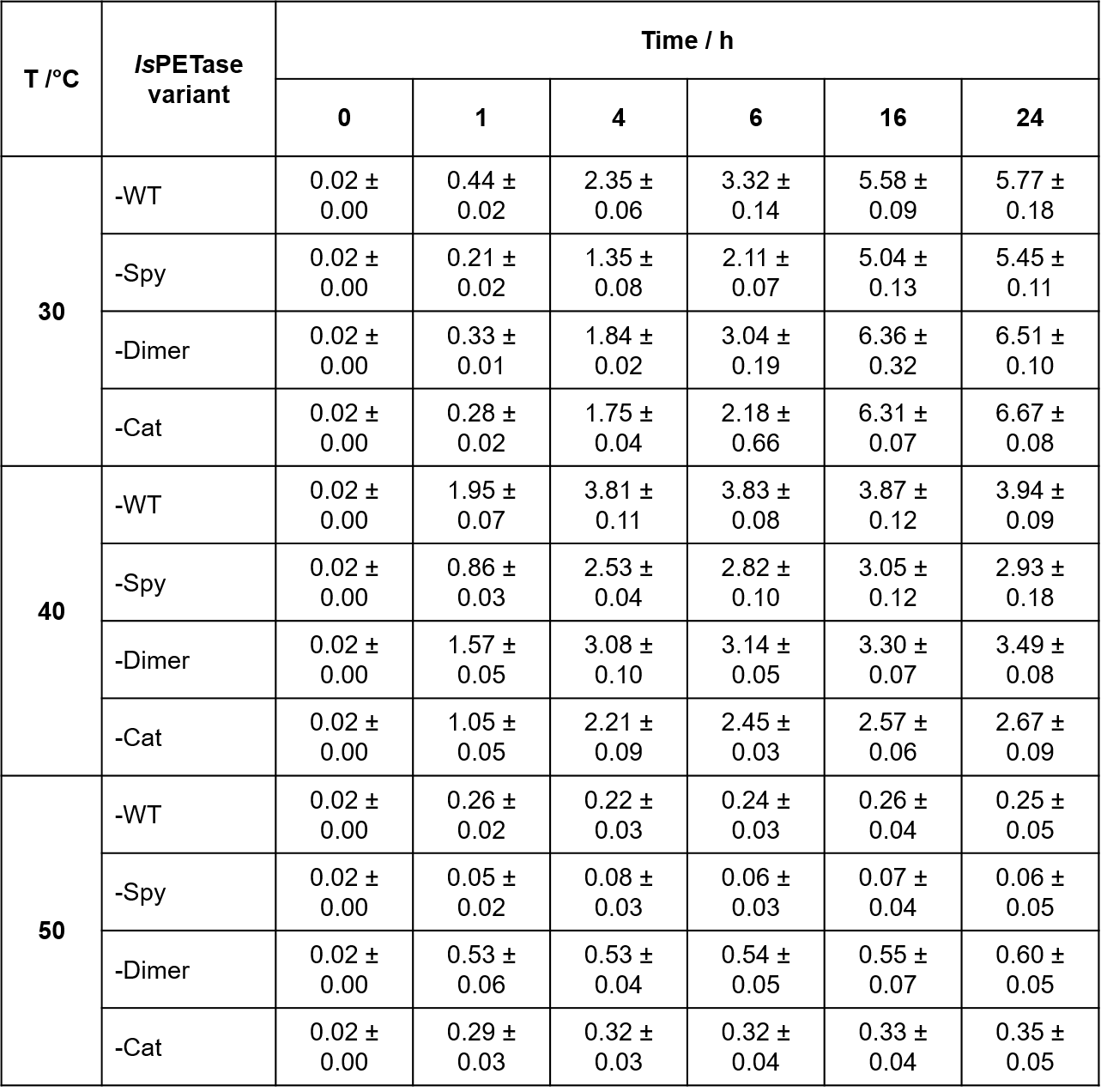


**Table S6.** PET degrading activities of the linear and cyclic *Is*PETase variants at increasing temperature at 1100 rpm. Soluble degradation product release monitored over 24 hours using the absorbance at 240 nm (mAU).


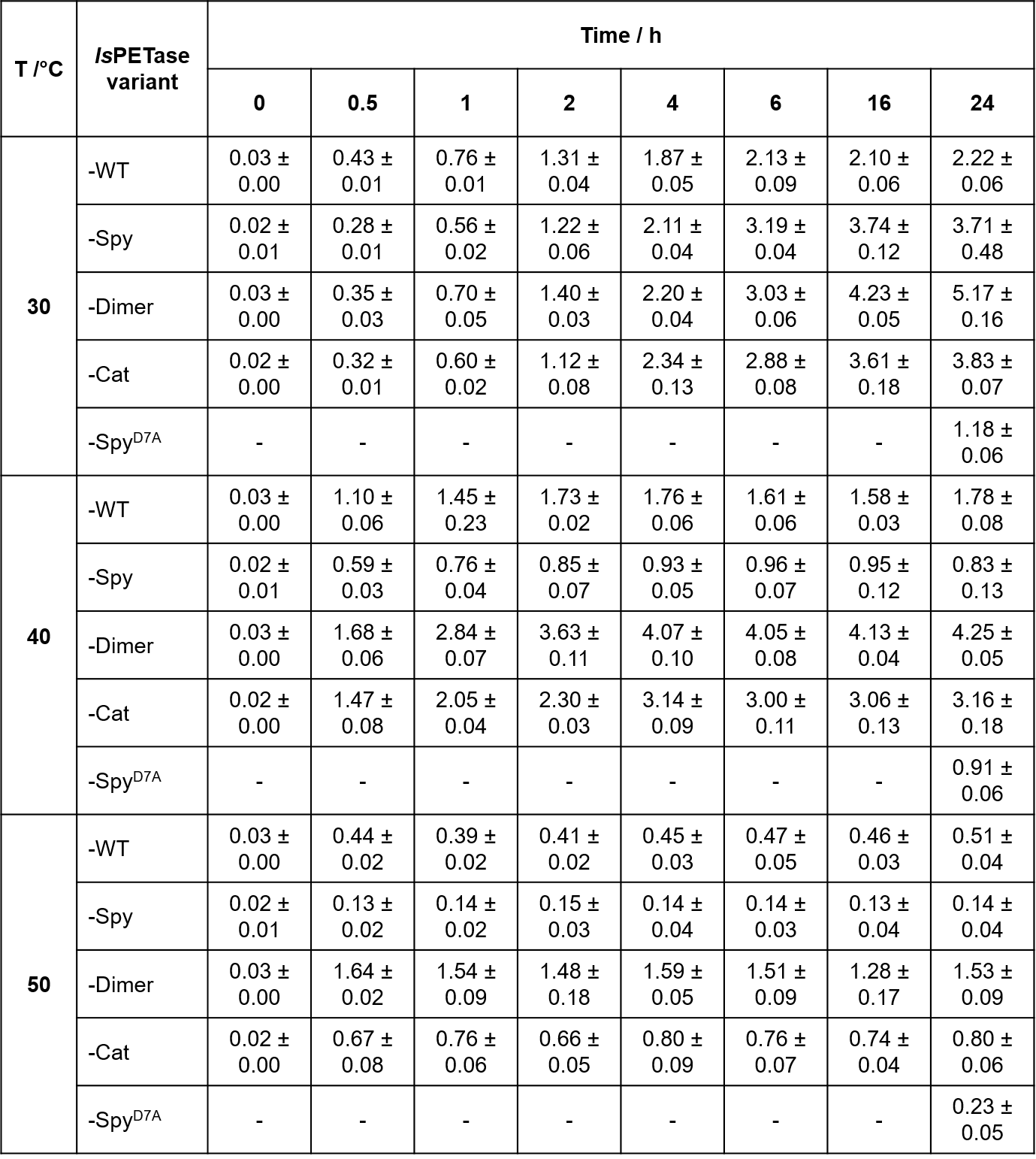


**Table S7.** PET degrading activities of the linear and cyclic DuraPETase variants at increasing temperature at 0 rpm. Soluble degradation product release monitored over 24 hours using the absorbance at 240 nm (mAU).


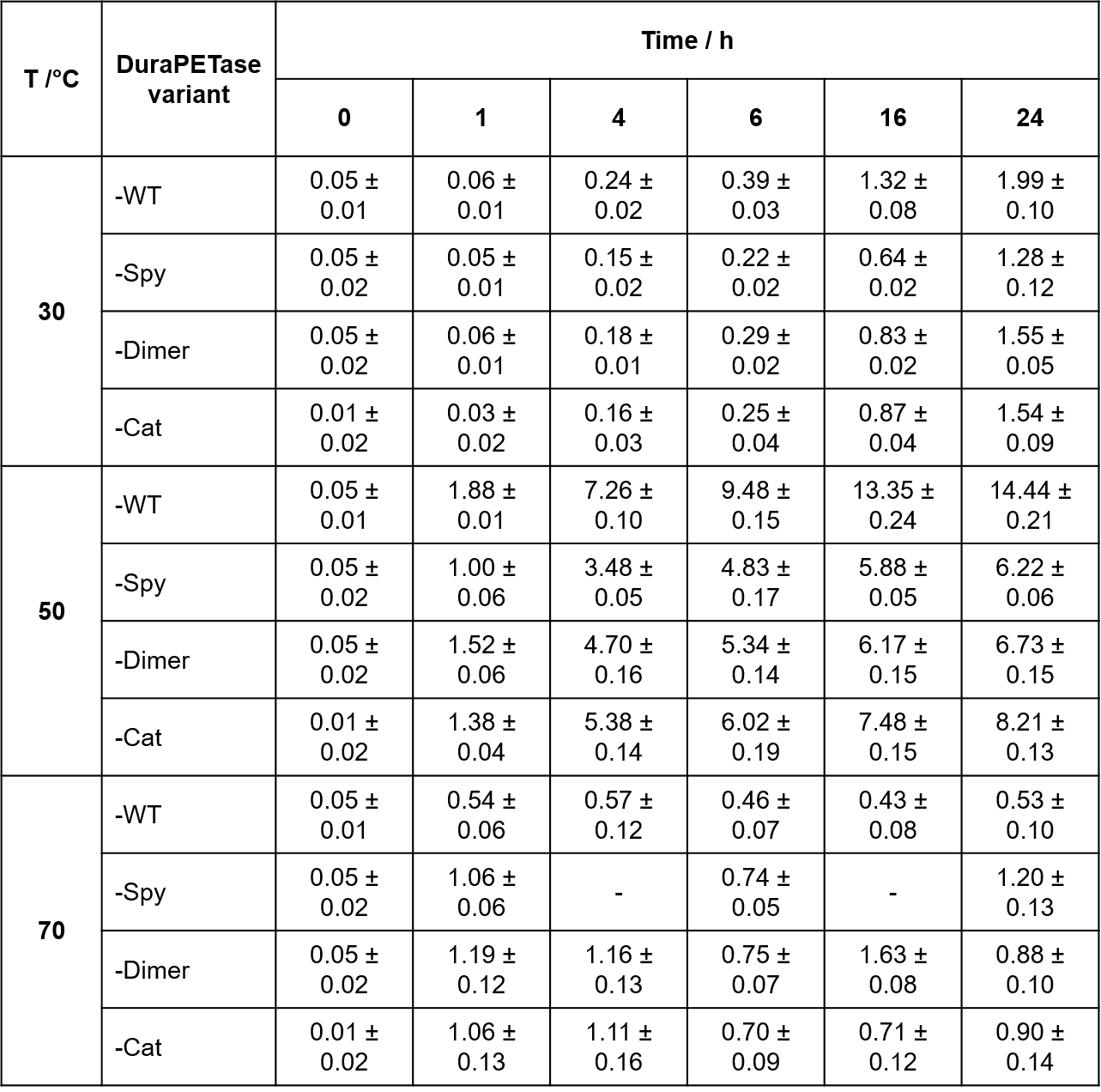


**Table S8.** PET degrading activities of the linear and cyclic DuraPETase variants at increasing temperature at 1100 rpm. Soluble degradation product release monitored over 24 hours using the absorbance at 240 nm (mAU).


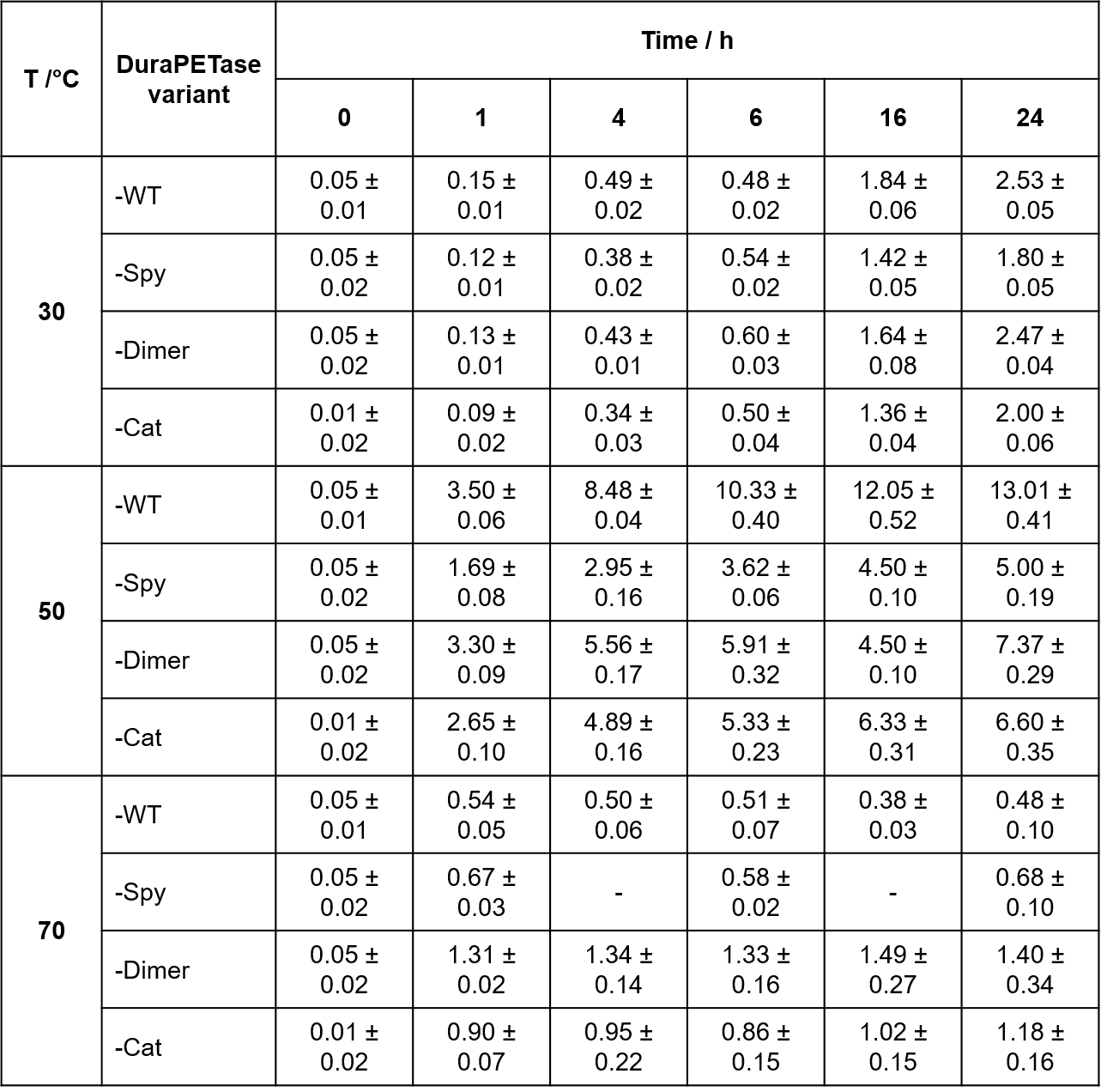


**Table S9.** Gene sequences of the *Is*PETase variants and DuraPETase (5’ to 3’). The *Is*PETase N-terminal signal peptide sequence was removed to enhance recombinant expression.

**
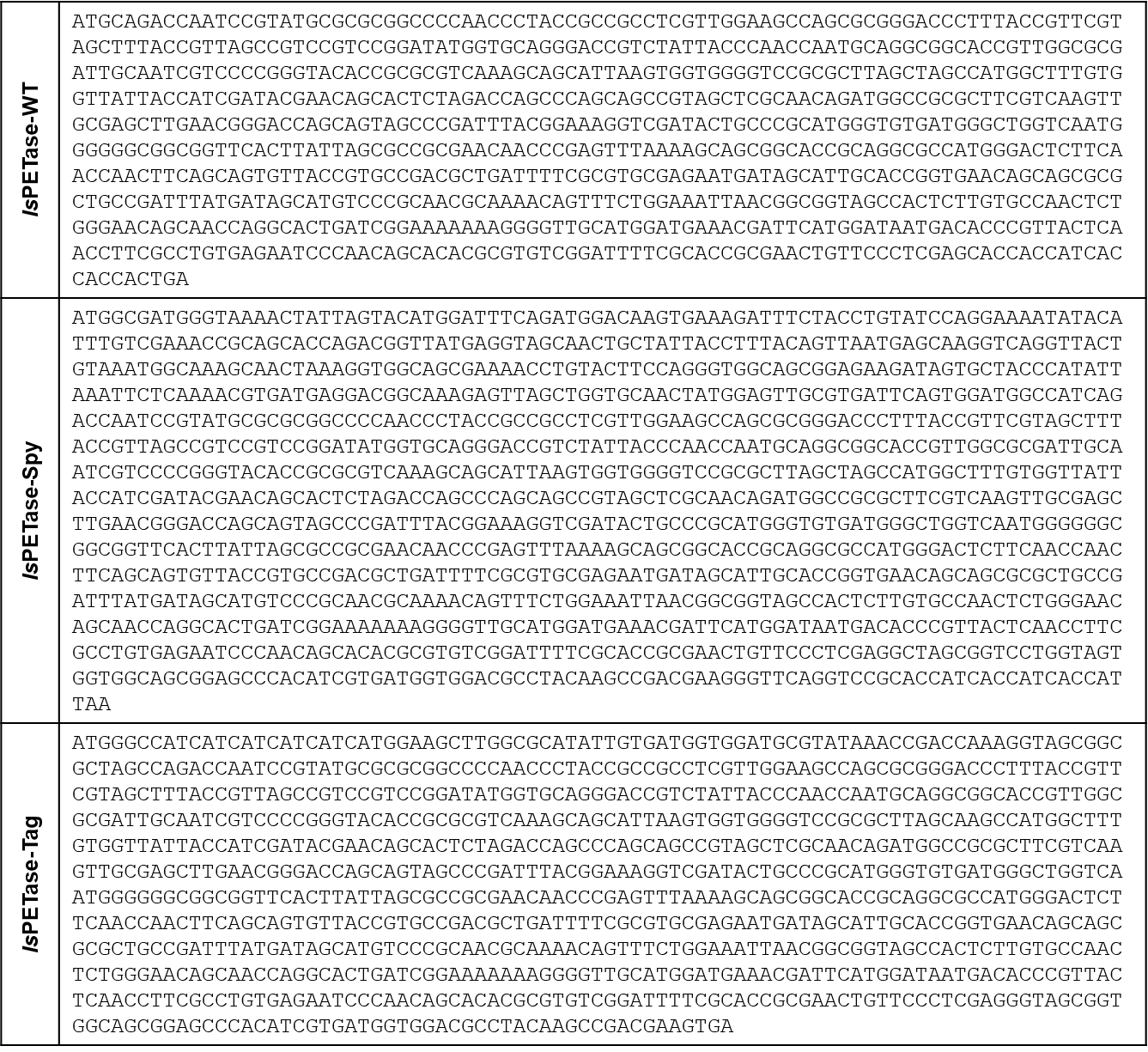
**

**
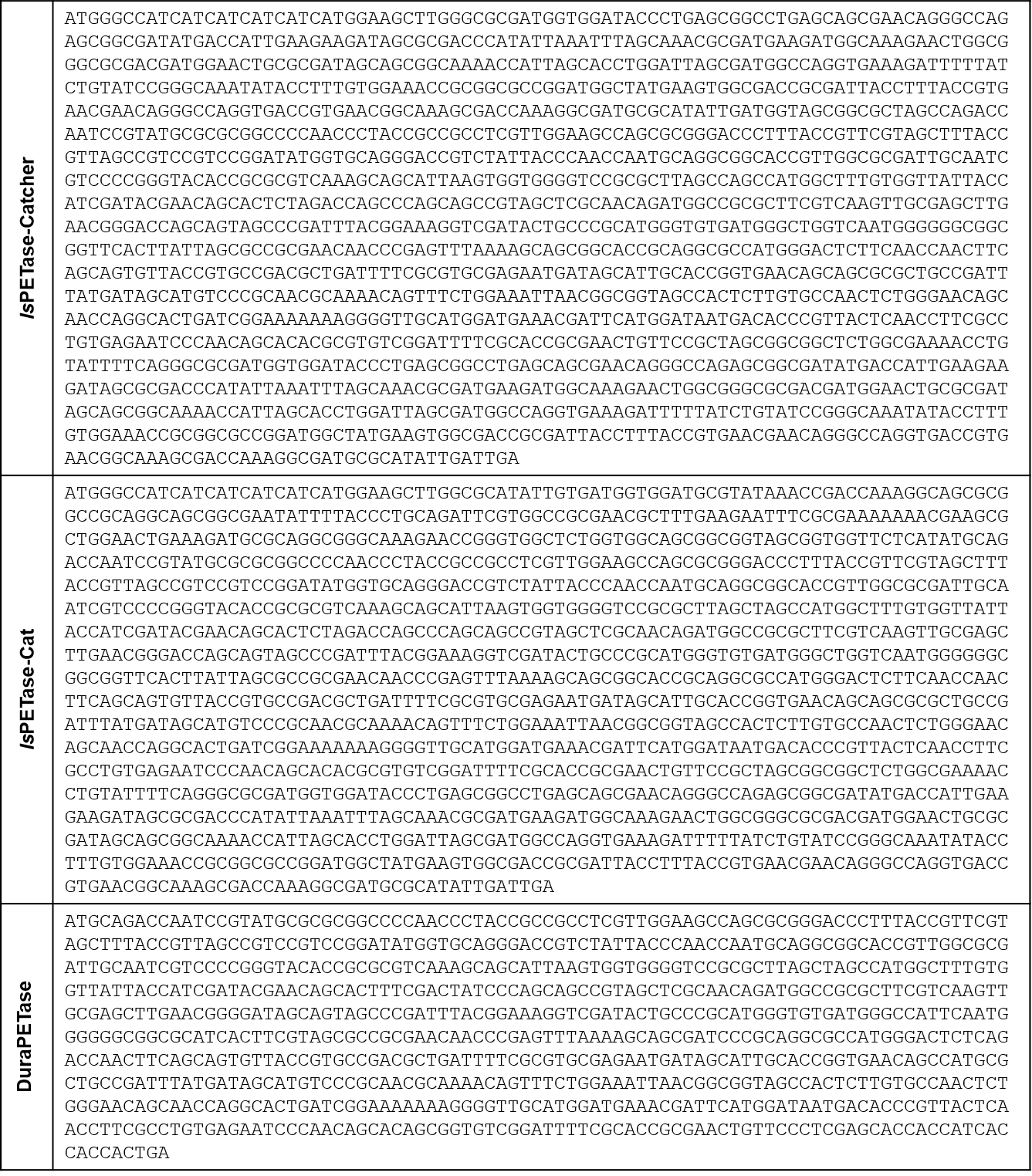
**

**Table S10.** Amino acid sequences of the *Is*PETase variants and DuraPETase. The *Is*PETase N-terminal signal peptide sequence (26 amino acid residues) was removed to enhance recombinant expression.

**
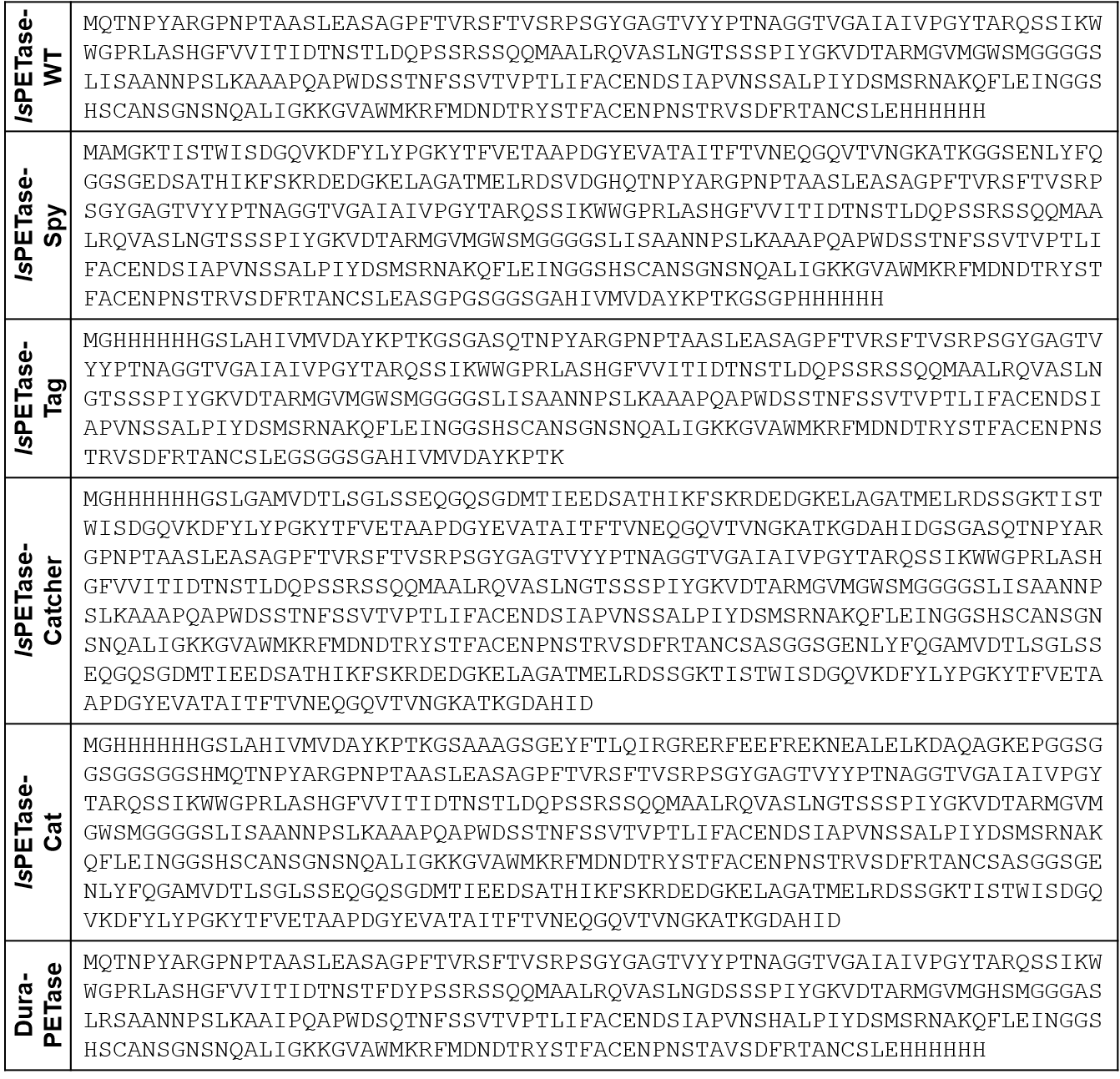
**

**Table S11.** Forward (F) and reverse (R) primers used in this study for SDM.


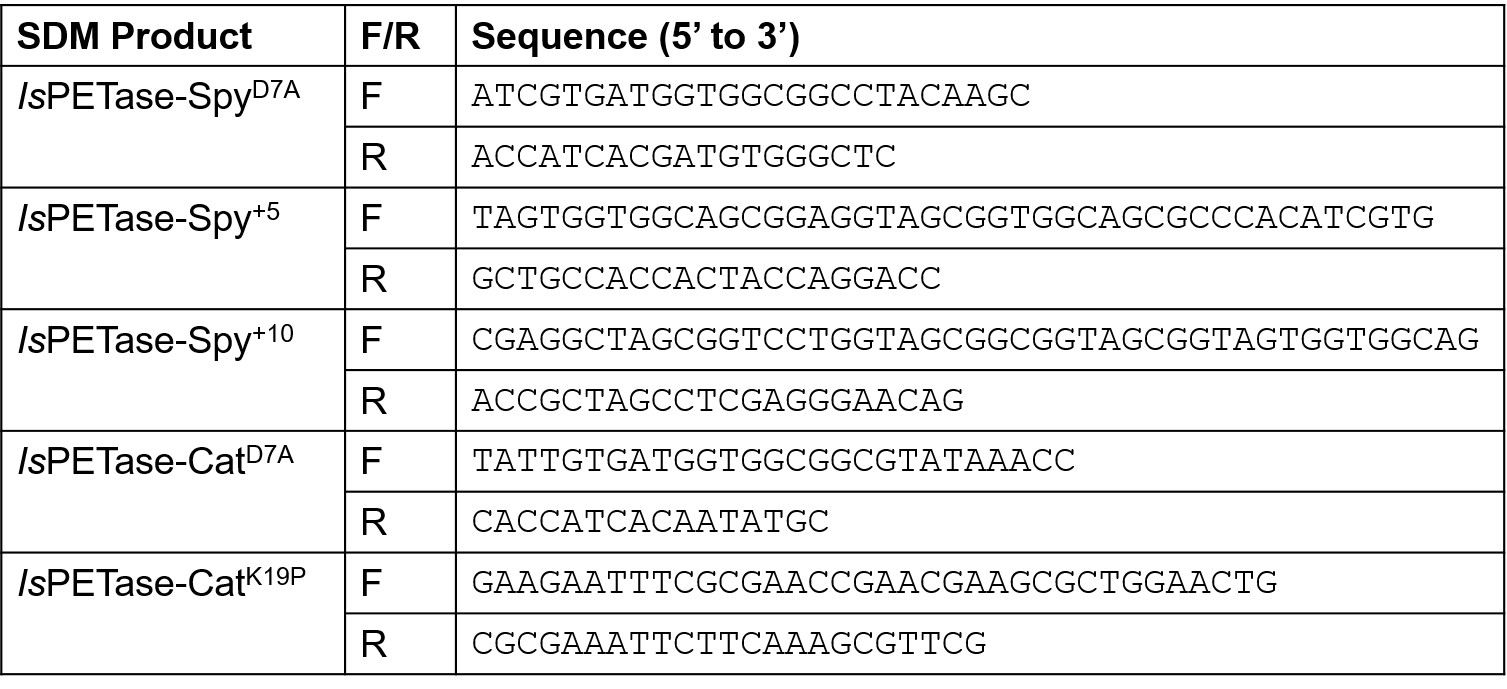

Supplement: Supplementary file 1 — Supplementary Information. [file 41598_2023_27780_MOESM1_ESM.docx]
